# Mitochondrial transcript abundance in breast cancer circulating tumour cells may reflect both blood-derived transcripts and biological heterogeneity

**DOI:** 10.1007/s10585-026-10430-0

**Published:** 2026-10-05

**Authors:** Alicja Staśczak, Agnieszka Stankiewicz, Justyna Topa, Zofia Piesik, Anna Żaczek, Sebastian Student, Tomasz Stokowy, Aleksandra Markiewicz

**Affiliations:** 1 Department of Systems Biology and Engineering, Faculty of Automatic Control, Electronics and Computer Science, Silesian University of Technology, Akademicka 14 Str, 44-100 Gliwice, Poland; 2 Biotechnology Centre, Silesian University of Technology, Akademicka 2a Str, 44-100 Gliwice, Poland; 3 Laboratory of Translational Oncology, Intercollegiate Faculty of Biotechnology, University of Gdansk and Medical University of Gdańsk, Debinki 1 Str, Gdańsk, 80-211 Poland; 4 Institute of Informatics, Faculty of Mathematics, Physics and Informatics, University of Gdańsk, Wita Stwosza 57 Str, 80-952 Gdańsk, Poland; 5 Scientific Computing Group, IT Division, University of Bergen, Nygårdsgaten 5 Str, Bergen, 5015 Norway; 6 Interdisciplinary Pomeranian Centre for Digital Medicine, Medical University of Gdańsk, Debinki 7 Str, 80-210 Gdańsk, Poland

**Keywords:** Liquid biopsy, Mitochondrial reads, Platelets, RNA-seq, Single-cell

## Abstract

Circulating tumour cells (CTCs) are rare cells frequently profiled on a single cell level. A key indicator of single-cell transcriptome quality is the percentage of reads encoded by genes in the mitochondrial genome (MT-reads). No systematic data exists on MT-reads content in CTCs, making it unclear whether this parameter can reliably indicate CTCs’ transcriptome quality. We collected RNA-Seq profiles of 349 single breast cancer (BC) CTCs and 129 white blood cells (WBCs) enriched with four methods to assess the level of MT-reads and the expression of markers of the most abundant blood components—red blood cells (RBCs) and platelets (PLTs). The median percentage of PLTs and RBCs transcripts in CTCs was 2% (0–38%) and 0.8% (0.0006-84%), respectively, with significant differences between enrichment methods (*p* < 2.2^−16^). As a result, 44% of CTCs showed MT-reads level exceeding 10%, commonly considered a threshold indicating low-quality cells. Level of MT-reads in CTCs was frequently positively correlated with PLTs and RBCs markers; though in selected datasets increased MT-reads correlated with higher expression of oxidative phosphorylation and mitochondria biogenesis genes. Isolated CTCs themselves showed varied degree of cancer cells characteristics depending on the applied criterion (BC and WBC marker levels, aneuploidy score), underlining the challenges in CTCs identification. To conclude, the proportion of MT-reads in single CTCs is not a reliable standalone indicator of CTCs’ transcriptome. Elevated MT-read fraction in CTCs may arise from multiple factors, including technical aspects of blood sample processing, potential contributions of platelet- and erythroid-derived transcripts or intrinsic biological processes occurring in CTCs.

**Graphical abstract:**

**Figure:**
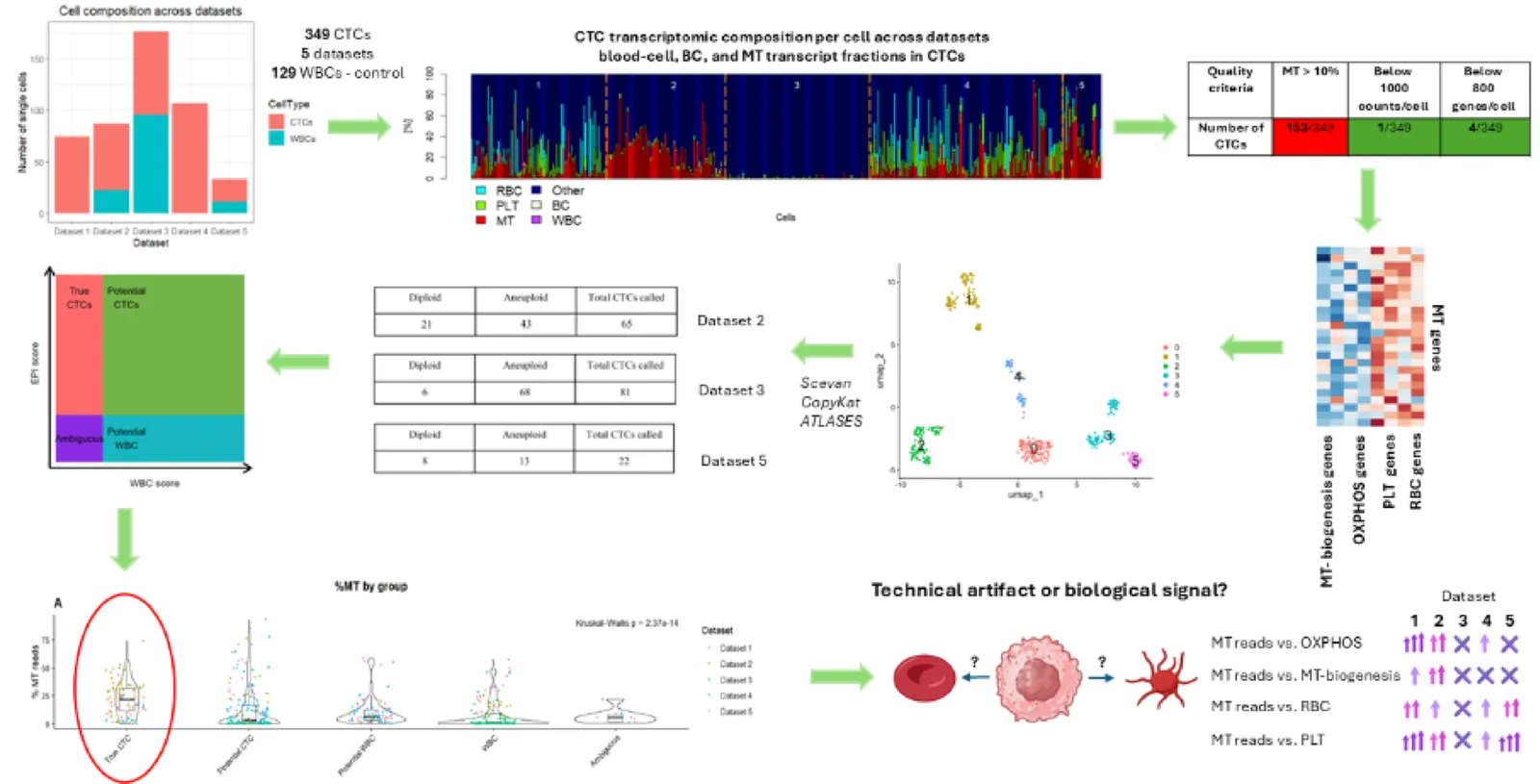

**Supplementary Information:**

The online version contains supplementary material available at https://doi.org/10.1007/s10585-026-10430-0.

## Introduction

Breast cancer (BC) is the most frequently diagnosed cancer type in women worldwide, with 2.26 million new cases in 2020 and the leading cause of cancer-related deaths [1]. During metastatic progression cancer cells from the solid tumour intravasate into lymphatic or blood vessels, becoming circulating tumour cells (CTCs) that can initiate metastases at distant sites [2]. The detection of CTCs in peripheral blood, known as liquid biopsy, is a promising tool for non-invasive cancer analysis—developing clinically valid prognostic and predictive markers for monitoring treatment response and detecting minimal residual disease [3, 4]. Various methods have been developed to capture CTCs [5, 6], they can be broadly categorized as marker-dependent (capturing cells positive for a cancer marker or enriching cells lacking blood cell markers) or marker-independent, relying on various physical properties of CTCs (e.g., larger size, decreased deformability, or specific density) that distinguish them from surrounding blood cells. Nevertheless, CTCs isolation remains challenging due to CTCs’ rarity, heterogeneity [7–9] and short half-life in blood, estimated at 1–2.4 h [10]. This brief viability window makes it difficult to obtain a high fraction of good-quality cells for in-depth transcriptomic profiling. One of the parameters for the identification of low-quality cells in single-cell RNA sequencing (scRNA-seq) is the percentage of reads mapped to mitochondrially-encoded genes (MT-reads) [11–14]. Elevated MT-reads (in the literature often referred to as ‘mtDNA%’) can indicate stressed and apoptotic cells [15, 16], but can also result from cell membrane damage and cytoplasmic transcript leakage from the cell [11]. The proposed threshold for identifying human cells with low-quality transcriptomes is 10% MT-reads content [12]. However, the number of mitochondria vary significantly across different tissues due to their physiological characteristics, such as metabolic activity [13, 17]. Currently, there is a lack of systematic data regarding MT-reads content in CTCs. Consequently, little is known about the applicability of the percentage of MT-reads as a quality control metric for CTCs transcriptomes. CTCs represent a unique sample type—they are cells originating from epithelial tissue that circulate in blood, a foreign niche, where they are exposed to different factors than in their native environment. Platelets (PLTs) have been shown to shield CTCs from blood-related environmental stress by mitigating the effects of shear forces [18] and protecting cells from natural killer cell attacks [19, 20]. PLTs and red blood cells (RBCs) are the most numerous morphotic elements of blood. There are approximately 5 × 10^9^ RBCs per millilitre of blood and 1.5–4.5 × 10^8^ PLTs per millilitre of blood [21, 22]. Interestingly, high content of MT-reads (among the 25 most highly expressed genes) was shown in both RBCs, RBC-derived extracellular vessicles (EVs) [23] and PLTs [24, 25]. At least for the latter, it is unsurprising since mitochondria are indispensable for PLTs [26], whose functioning is highly energy-dependent [27]. CTCs may interact with RBCs [28] and PLTs [18, 29] directly or by taking up PLT-derived EVs [30]. Studies on PLTs revealed that they are capable of releasing both free and EV-encapsulated mitochondria [31, 32]. Moreover, cancer cells were shown to acquire PLT-derived mitochondria, which equipped them with more invasive properties [32, 33].

In this study, we conducted a comprehensive evaluation of the transcriptomic composition of BC patients’ CTCs enriched using different platforms: CTC-iChip, Parsortix^TM^, ClearCell^®^ FX, and CD45 Dynabeads depletion. Our analysis focused particularly on MT-reads, as well as markers of PLTs and RBCs, whose expression has previously been reported in liquid biopsy samples such as CTCs [34, 35], disseminated cancer cells (DTCs [36, 37]) and white blood cells (WBCs) [38]. We aimed to investigate the possible origin of the MT-reads in CTCs beyond their conventional use as an indicator of cell transcriptome quality. We hypothesized that the proportion of MT-reads in CTCs might not always reflect the low quality of a given cell, but also indicate intrinsic biological processes in CTCs, interactions between CTCs and other blood components or contamination of rare CTCs transcriptomes by transcripts from abundant circulating cells. This approach aims to provide a more nuanced understanding of single CTCs transcriptomes and the challenges associated with their isolation, analysis and data interpretation.

## Materials and methods

### Selection of datasets and initial processing

Publicly available datasets containing well-annotated transcriptomic profiles of CTCs (and accompanying WBCs from the same study when available) from BC patients were chosen. We manually selected datasets that contained information on the method of CTCs enrichment, staining and CTCs detection strategy, single cell isolation method, reverse transcription and preamplification protocol, whether obtained cells represented a pool/single cell or cell cluster (homotypic or heterotypic) and included only those representing single CTCs or WBCs. The final CTCs dataset was compiled from five sources (described in 3.1 Section): GSE75367 [39], GSE109761 [40], GSE210651, GSE186288 [41], GSE143626 [42], and our dataset GSE268201, which is a result of research approved by the Bioethics Committee of the Medical University of Gdańsk (approval number NKBBN/433/2017 and NKBBN/748/2019–2020); analyses were performed in accordance with institutional guidelines and regulations, informed consent has been obtained from all patients included in our study cohort and samples processing was in details described in our previous work [43]. Summary of the CTCs enrichment protocols used in the five datasets is presented in the Supplementary document1.

WBCs included in the three datasets were derived from two sources—in dataset 2 (GSE109761) and dataset 5 (GSE268201), WBCs were obtained from BC patients, although not necessarily from the exact same individuals from whom CTCs were collected; importantly, these WBCs were enriched and processed using the same protocol as applied to CTCs. In dataset 3 (GSE210651, GSE186288), CD45^+^ cells from healthy donors were isolated as single cells using a microfluidic platform (C1 Fluidigm), similar to that used for CTCs enrichment, and subsequently processed following the same downstream workflow as CTCs samples. The clinical status of patients differed across the datasets. Dataset 1 included CTCs from metastatic BC patients who had received multiple lines of therapy. Dataset 2 reported including patients with invasive BC with high tumour load, who discontinued treatment due to disease progression. Treatment and stage of the disease were not reported for datasets 3 and 4. Dataset 5 consisted predominantly of non-metastatic, treatment-naïve patients (11 non-metastatic patients, of which 10 were untreated; 1 untreated metastatic patient; 1 patient with unknown treatment and metastatic status). The original studies applied different transcriptome quality-control criteria before downstream analyses. Datasets 1 excluded cells with fewer than 100000 mapped reads. Dataset 2 removed cells with fewer than 800 detected genes (≥ 1 mapped read) and cells showing substantial stromal gene expression in the absence of cancer-associated genes (cut-off and gene not specified). Dataset 3 excluded cells with fewer than 50000 total reads or fewer than 1 500 expressed genes. Dataset 4 excluded cells with fewer than 100000 uniquely mapped reads and cells expressing CD45 or CD16 above the specified threshold (log_10_ (RPM + 1) > 0.4). Dataset 5 did not apply transcriptome quality filtering prior to analysis; cells’ were tested by multiplex PCR before library preparation to select those with good quality transcriptome (described in [43]). Additionally 7 PLT pool samples processed *via* scRNA-seq (GSE109761 [40]), as well as 4 RBC samples and their EVs sequenced *via* bulk RNA-seq (GSE108378) [23] were included.

HISAT2 (2.2.1 version) was used to reads mapping alignment to GRCh38.p7 human genome reference [44]. Annotations were performed based on GENCODE (version 26) [45]. Read counting per gene was conducted using featureCounts [46]. The data was merged into a single matrix, where columns represent cells and rows correspond to individual genes. Cell names adhere to those originally used in publicly available datasets, with a cell classification appended to each name (e.g., ‘CTC’ indicating a circulating tumour cell) and a designation of the dataset source using the first three letters of the first author’s surname in the provided dataset.

### Selected lists of characteristic markers genes

For a detailed analysis of single-cell gene expression levels, characteristic marker groups were identified for MT (37 genes), PLTs (177 genes), RBCs (139 genes), WBCs (18 genes), BC cells (17 genes; Supplementary Table 1). All 37 genes encoded in the MT genome were included [47]. The identification of genes in the RBC and PLT marker groups was based on lists provided by the Human Protein Atlas, specifically Cluster 1: Erythroid cells—Oxygen transport and Cluster 57: Platelets—Hemostasis [48]. Additionally, to the PLT marker group we added 20 manually curated genes. These were genes with the highest expression in PLT pools from the control dataset [40] but with following exclusion criteria: MT genes, RBC-specific genes according to Kerkelä et al. [23], whole blood-specific genes according to Xie et al. [24] and genes encoding proteins of ribosomal subunits. The BC marker group included genes frequently used to detect CTCs in BC patients [49–52], while WBCs markers were selected based on commonly used genes for various immune cell populations [34]. Subsequently, the percentage of reads for each class (RBCs/PLTs/WBCs/MT/BC) relative to the total reads per cell was calculated. The remaining reads, not belonging to the RBC/PLT/WBC/MT/BC classes were grouped into the “Other” category and encompassed all mapped reads, including non-protein-coding genes.

### Statistical and bioinformatics analysis

The computational analyses were performed using R programming language (R-4.3.2)/Bioconductor [53]. Data normalization utilized the ComputeSumFactors method [54], available in the scran package (1.18.7 version). For the RBCs bulk RNA-seq dataset, standard normalization from the DESeq2 (1.42.1 version) package was applied [55]. No cells were excluded based on MT-reads fraction, as mitochondrial abundance constituted the primary outcome of the study. Original datasets were reprocessed without applying an MT-based quality filter. Comparative analyses included fold change (FC) estimations and Student’s *t*-test calculations with significance thresholds set as *p* < 0.05 and false discover rate (FDR) < 0.05, and log_2_FC, as applied in our other publications [56]. Genes with median log_2_FC ≤ −1 and log_2_FC ≥ 1 were considered down-regulated and up-regulated, respectively. To estimate contamination levels of CTCs and WBCs transcriptomes, the DecontX (1.2.0 version) algorithm was applied [57] independently to each dataset using the default parameters. DecontX is a method based on a Bayesian approach used to detect and eliminate contaminants in individual cells. It assumes that the observed cell expression is a mixture of counts from two multinomial distributions: counts of native transcripts from the actual cell population and counts of contaminating transcripts from other cell populations included in the analysis. Potential cell doublets were identified using scDblFinder (1.18.0 version) [58] with the default parameters settings. Doublet detection was performed separately for each dataset whenever the number of cells was sufficient. For smaller datasets, in which reliable dataset-specific estimation was not feasible, a global reference including all cells was used. Graphics were created using the R’s graphics package and Matplotlib [59]. Gene Set Enrichment Analysis (GSEA) was performed using the HALLMARK_APOPTOSIS gene set to assess differences in apoptosis-related transcriptional activity between cells with high (> 10%) and low (≤ 10%) MT-reads fractions. Analyses were conducted separately for CTCs within individual datasets and for WBCs, with enrichment evaluated based on the normalized enrichment score (NES) and FDR-adjusted q-value. Apoptosis module score was calculated based on the expression of eight pro-apoptotic genes (*BBC3*,* PMAIP1*,* BCL2L11*,* TNFRSF10B*,* FAS*,* BAX*,* CASP3*, and *CASP7*) and compared between cells with high (> 10%) and low (≤ 10%) MT-reads fractions. Differences in apoptosis module scores were assessed separately for CTCs and WBCs using the Wilcoxon rank-sum test.

The normalized expression matrix was transferred into the Seurat object as the data layer. Dimensionality reduction and dataset integration were performed using the standard Seurat workflow [60], including identification of highly variable genes (FindVariableFeatures, 2000 genes, vst method), data scaling (ScaleData), and principal component analysis (RunPCA, 30 components). Batch and dataset-specific effects were corrected using Harmony integration [61] with dataset ID as the batch variable. Based on the Harmony embedding, a shared nearest-neighbor graph was constructed (FindNeighbors), followed by unsupervised clustering (FindClusters) and computation of a two-dimensional UMAP projection (RunUMAP). UMAP was calculated on the Harmony-corrected embedding using the first 30 Harmony dimensions.

Module scores (RBC, WBC, PLT, MT, and EPI) were calculated using the Seurat AddModuleScore function on log-normalized expression values. Narrower marker gene sets were defined for each module using the following genes: EPI score—*CDH1*,* EpCAM*,* KRT5*,* KRT6A*,* KRT6B*,* KRT8*,* KRT14*,* KRT18*,* KRT19*; WBC score—*PTPRC*,* CD3D*,* CD14*,* CD19*,* GZMA;* RBC score—*HBB*,* HBA1*,* HBA2;* PLT score—*NAP1L1*,* TLN1;* MT score—all 37 genes encoded in MT-DNA. To further characterize cells originally isolated and annotated as CTCs in all five datasets, cells were subdivided into four groups based on epithelial (EPI score) and leukocyte (WBC score) module score distributions. CTCs stratification was performed using score quartiles (25th and 75th percentiles), allowing separation of cells with high versus low EPI and WBC transcriptomic scores. Four categories were defined: (i) ‘true CTCs’—cells with mid-to-high epithelial signal (EPI score > Q1) and low leukocyte signal (WBC score ≤ Q1); (ii) ‘potential CTCs’—cells with mid-to-high epithelial signal (EPI score > Q1) and mid-to-high leukocyte signal (WBC score > Q1); (iii) ‘potential WBCs’—cells with low epithelial signal (EPI score ≤ Q1) and mid-to-high leukocyte signal (WBC score > Q1); (iv) ‘other/ambiguous cells’ that did not meet the above criteria (EPI score ≤ Q1 and WBC ≤ Q1). Cells isolated and annotated as WBC in the original dataset were labelled as such.

To assess large-scale chromosomal copy number alterations (CNVs) and support discrimination between aberrant and non-aberrant cell populations, two independent CNV inference approaches were applied to the single-cell RNA-seq data: CopyKAT [62] and SCEVAN [63]. CopyKAT was performed on three datasets in which a matched WBC population was available as a diploid reference. In these analyses, WBCs were explicitly used as the baseline control, enabling robust signal normalization and classification of cells as diploid or aneuploid relative to the healthy immune cells compartment. In parallel, CNV inference was conducted using SCEVAN in a reference-free mode. In this setting, no external control population was provided, and diploid-like cells were identified internally based on the intrinsic structure of the transcriptomic data.

To independently check cell identity and evaluate transcriptomic similarity between cells originally isolated and annotated CTCs or WBCs, single-cell RNA-seq profiles were projected onto reference cell atlases using SingleR [64] together with datasets from the celldex package, including the HumanPrimaryCellAtlas (HPCA) [65] (a broad reference of primary human cell types used for general classification) and Blueprint/ENCODE [64, 66, 67] (a hematopoietic and peripheral blood atlas supporting identification of blood-derived populations).

## Results

### Collected datasets

To compare different datasets and evaluate the performance of CTCs isolation methods from BC patients, we re-analysed four publicly available datasets (criteria specified in 2.1 Section) and one novel dataset recently generated in our laboratory (GSE268201 [43]). The integrated collection consisted of 478 single cells: 349—CTCs and 129 WBCs (referred to as CD45 cells or normal cells—NCs in the original studies). Importantly, cells originated from various BC stages, subtypes, treatment conditions and were harvested using different methods and protocols; pre-analytical steps of samples collection analysis were not always reported in the original manuscripts, which represents a limitation for the data interpretation. Detailed information regarding the collected and integrated single-cell data are summarized in Table 1.

**Table 1:** List of collected and analysed datasets (1 [39], 2 [40], 3 [41], 4 [42], 5 [43]) used in the analysis along with individual features

Dataset	1 (Jordan et al. [39]) GSE75367	2 (Szczerba et al. [40]) GSE109761	3 (Poonia et al. [41]) GSE210651, GSE186288	4 (Ebright et al. [42]) GSE143626	5 (Topa et al. [43]) GSE268201
Total number of cells	74	87	177	107	33
Number of CTCs	74	65	81	107	22
Number of WBCs	–	22	96	–	11
Breast cancer subtype	ER+/HER2-, TNBC	Divided into ER/PR/HER2	TNBC, ER+/PR+/HER2-, ER-/PR-/HER2+	ER and/or PR+	ER and/or PR + as well as HER2+
Number of srr files	148	145	177	658	33
Sequencing strategy	Paired-end	Single-end	Paired-end	Single-end	Paired-end
Platform	Illumina	Illumina	Illumina	ABI_SOLID	Illumina
Instrument	Illumina HiSeq 2000	Illumina NextSeq 500	Illumina MiSeq, NextSeq 500	AB 5500xl Genetic Analyzer	Illumina MiniSeq, HiSeq X Ten System
Blood sample collection	Inf. not provided	EDTA-tube	EDTA-tube	Inf. not provided	EDTA-tube
Patients	Metastatic, treated	Patients with high tumour load, treatment discontinued	Inf. not provided	Inf. not provided	Majority non-metastatic and not treated
CTC enrichment method	Microfluidic CTC-iChip	Microfluidic Parsortix^TM^, 6,5 μm Cassette	Microfluidic Integrated ClearCell^®^ FX/Polaris^TM^ workflow	Microfluidic CTC-iChip	Dynabeads^TM^ CD45-depletion
CTC detection markers	EpCAM+, HER2+, CD45-	EpCAM+, HER2+, EGFR+, CD45-	Calcein AM+, CellTracker Orange + CD45-, CD31-	EpCAM+, Cadherin 11+, HER2+, CD45-, CD14-, CD16-	EpCAM+, E-Cadherin+, csVIM+, MCAM+, CD45-, CD31-
Method of single cells collectioN	Micromanipulation (Eppendorf TransferMan)	Micromanipulation (CellCelector, ALS)	Cell isolated on Polaris^TM^ capture sites	Micromanipulation (Eppendorf TransferMan)	Micromanipulation (Eppendorf TransferMan)
RNA preparation method	SMARTer Ultra Low Input Kit for RNA v3 (Clontech)	Smart-seq2	SMARTer^®^ Ultra^®^ Low RNA Kit for Illumina^®^ (Clontech)	SMARTer Ultra Low Input Kit for RNA v3 (Clontech)	Smart-seq2
Library construction	Nextera XT (Illumina)	Nextera XT (Illumina)	Nextera XT (Illumina)	Nextera XT (Illumina)	Nexter XT (Illumina)

csVIM—cell surface vimentin, EGFR—epidermal growth factor receptor, EpCAM—epithelial cell adhesion molecule, ER—estrogen receptor, HER2—human epidermal growth factor receptor type 2, MCAM—melanoma cell adhesion molecule, PR—progesterone receptor, PTPRC —- protein tyrosine phosphatase receptor type C (CD45-encoding gene), TNBC—triple-negative breast cancer

### Gene expression patterns in single CTCs and WBCs as defined by the original datasets

**Fig. 1:**
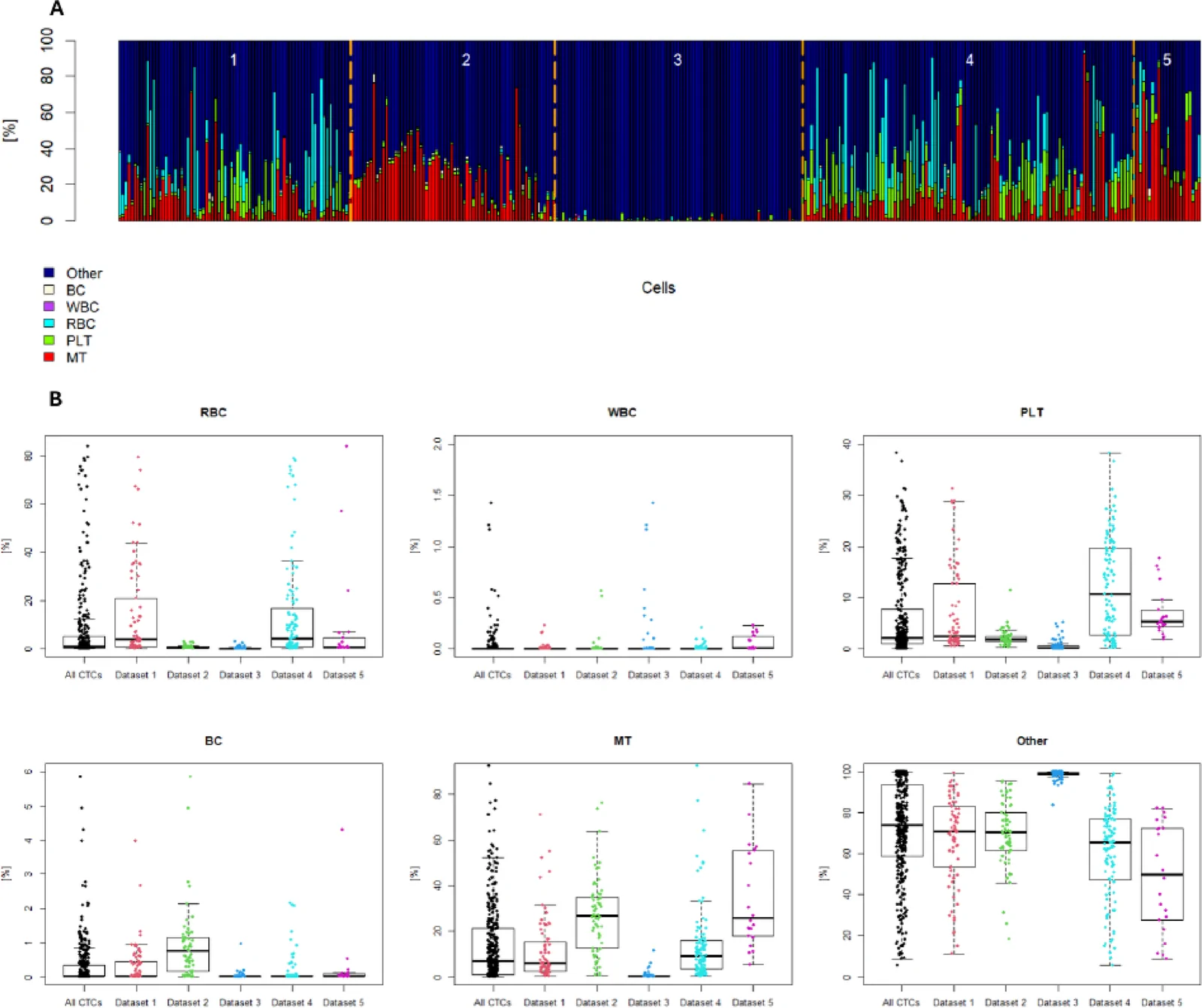
Transcriptomic composition of single CTCs according to classes of transcripts. Percentage of mapped reads in CTCs in relation to all counts in a given cell, according to transcript class. **A** percentage of mapped reads pertaining to BC, WBC, RBC, PLT markers, MT-encoded genes and remaining genes (Other) in the evaluated datasets; each bar corresponds to a single CTC. **B** summary percentage as in panel A grouped according to transcript types (RBCs, WBCs, PLTs, BC, MT, and Other); each dot represents a single CTC. Pairwise comparisons using the Wilcoxon rank sum test with continuity correction and Kruskal-Wallis test were performed, their exact values are presented in Supplemental Table 2

We analysed the composition of single CTCs and WBCs transcriptomes (as they were defined in the original manuscripts according to the given marker criteria, Table 1) with respect to the number of MT-reads, PLT, RBC, WBC, and BC marker reads in individual CTCs (Fig. 1A, B, statistics in Supplementary Table 2) and WBCs (Supplementary Fig. 1). Relatively high levels of MT-reads can be observed in CTCs isolated with almost all the methods, particularly in cells from dataset 2 (CTCs enriched with Parsortix) [40]. Only CTCs from ataset 3 [41], isolated by ClearCell^®^ FX/Polaris^TM^, are distinguished by a very small proportion of MT-reads, but also lower number of genes detected (Supplementary Table 3). WBCs displayed a similar pattern (Supplementary Fig. 1). PLT and RBC reads are present in almost all CTCs but are relatively high in CTCs from datasets 1, 4 (CTC-iChip isolated [39, 42]) and 5 (CD45 + cells depletion) [43]. Detailed summary statistics comparing datasets are provided in Supplementary Tables 2 and 3. The number of counts and genes expressed in each cell of all datasets is presented in Supplementary Fig. 2. Overall, ataset 3 [41], which included CTCs isolated via ClearCell^®^ FX/Polaris^TM^, contained single cells with the lowest proportion of MT-reads as well as PLT, RBC transcripts and the lowest number of genes detected. In turn, cells from dataset 2 (isolated with Parsortix [40]), exhibited the highest proportion of MT-reads and low levels of RBC and PLT transcripts. Analysis of the GSEA apoptotic signature in datasets 1, 2, and 4 (where division to two groups: MT-low and MT-high cells based on 10% cut-off was possible) showed borderline enrichment of apoptosis in CTCs from dataset 1 in MT-high cells (FDR = 0.083) and WBCs from ataset 2 (FDR = 0.079, but not CTCs from dataset 2; Supplementary Fig. 3A). Pro-apoptotic genes expression level (composed of *BBC3*,* PMAIP1*,* BCL2L11*,* TNFRSF10B*,* FAS*,* BAX*,* CASP3*,* CASP7*) performed separately for all CTCs and all WBCs showed that only in WBCs expression of pro-apoptotic genes is elevated (Supplementary Fig. 3B). Nevertheless, one needs to acknowledge the difficulty of assessing apoptosis based on transcriptomic level (especially in single cells), as apoptosis is significantly regulated on the protein level [14].

Analysed further, MT-reads fraction was positively correlated with the total normalized gene expression in CTCs and WBCs (Supplementary Fig. 3C). Moreover, transcriptome complexity was positively correlated with *MALAT1* expression—lncRNA retained in the nucleus, recently shown to be a good indicator of single-cell transcriptome quality ([68]; Supplementary Fig. 3D). At the same time *MALAT1* expression was higher in cells with higher MT-reads fraction (≥ 10%)—both in WBCs and CTCs (Supplementary Fig. 3E). Overall, this indicates that MT-reads fraction is not necessarily connected only with decreased transcriptome quality; though dataset-specific differences are observed—cells (CTCs and WBCs) from dataset 3 have the lowest expression of *MALAT1* and the lowest number of genes detected—Supplementary Table 3).

### Single cells transcriptome purity level

**Fig. 2:**
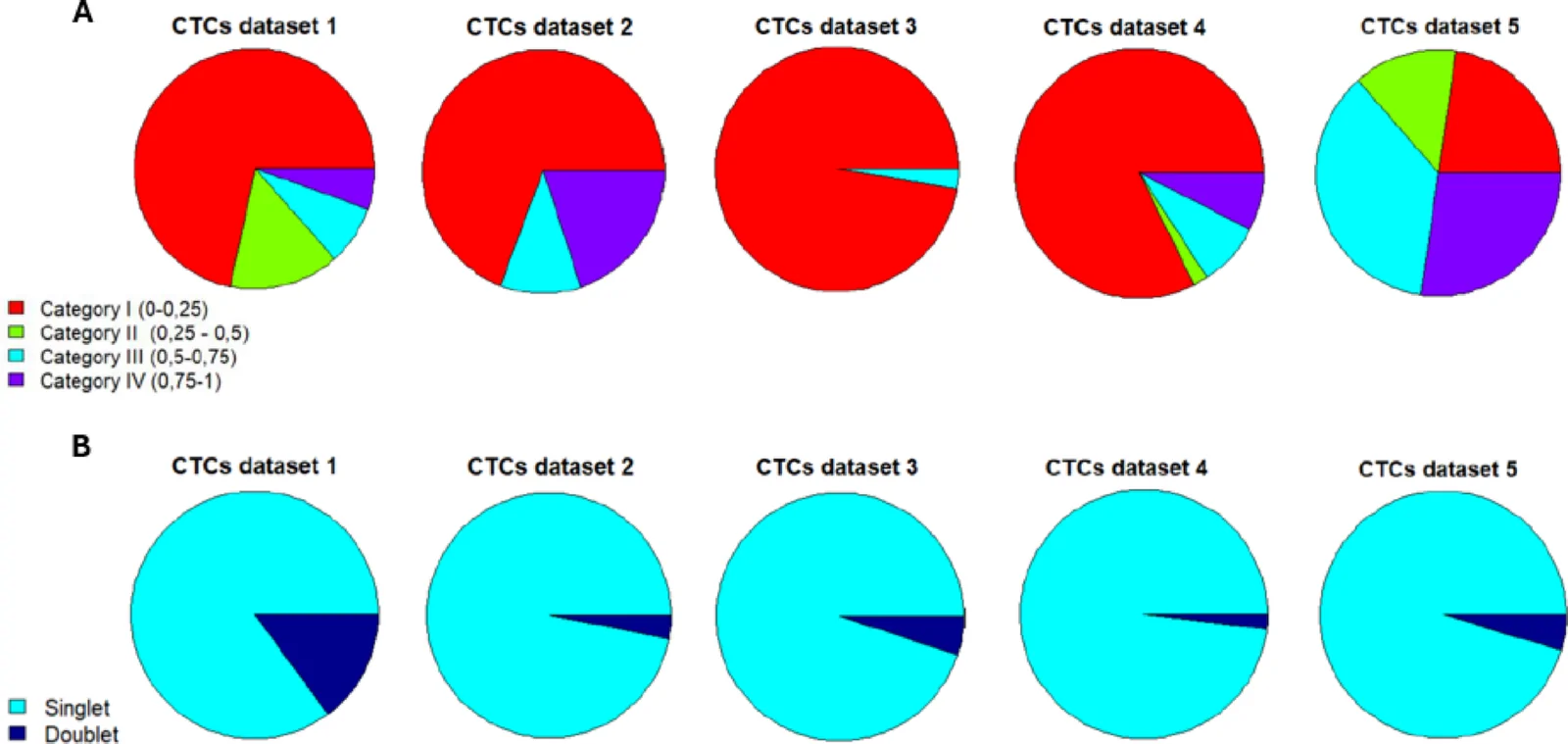
Assessment of CTC transcriptome purity and predicted doublet rates across the analysed datasets. **A** single CTCs’ transcriptome contamination level estimated using the DecontX in individual datasets; Category I-IV show purity from highest to lowest. **B** predicted proportion of singlets and doublets identified using scDblFinder in each dataset

DecontX was applied to group cells into four purity classes (as defined by DecontX) with progressively increasing contamination levels (Fig. 2A). CTCs isolated with microfluidics-based techniques showed the lowest level of contamination, with category I purity (contamination below 25%) detected in 72%, 69%, 98%, and 82% of the CTCs for atasets 1, 2, 3, and 4, respectively. In contrast, only 23% of cells belonged to category I purity were obtained using Dynabeads CD45-depletion (ataset 5). Differences in single-cell purity were smaller in the case of WBC subsets (Supplementary Fig. 1B), with over 75% of the cells in each dataset achieving purity class I, including WBCs isolated after Dynabeads CD45-depletion (dataset 5). The disparity between CTCs and WBCs purity levels could be due to genuine biological differences - CTCs’ increased uptake of transcripts (ambient RNA or transferred by vesicles) [69], expression of other cell lineage-markers [37, 70, 71] or from technically cleaner isolation of WBCs, which are more abundant in the sample and might be easier to collect than rare CTCs. It is important to note that DecontX score is not a direct evidence of contamination as it shows computational inferences of transcript admixture; it cannot distinguish technical carryover from biologically relevant transcript acquisition, endogenous expression or technical background (ambient RNA).

To assess if the observed contamination of CTCs and WBCs might result from accidental collection and sequencing of cells doublet, we applied scDblFinder [58]. T The predicted proportion of doublets among CTCs ranged from 1.87% in dataset 4–14.86% in dataset 1 in CTCs (Fig. 2B). As expected, both CTCs and WBCs classified as doublets showed higher DecontX contamination estimates than predicted singlets (data not shown). However, the relatively low frequency of predicted doublets and the lack of concordance between doublet rates and transcriptome purity across datasets indicate that doublets account for only a subset of cells with elevated contamination estimates and are unlikely to fully explain the differences in transcriptome purity observed across datasets. Assuming that ambient RNA level is comparable in single cells process according to the same protocol, WBCs purity can be taken as a basal reference level to which CTCs’ transcriptome purity can be referred. In such case, direct comparison can be made for datasets 2 and 5 when CTCs and WBCs were collected according to the same protocols. In both cases, DecontX purity levels were lower in CTCs than in WBCs. This could imply that what is detected in CTCs as contamination by DecontX, might be biologically relevant—either transcripts more efficiently taken up by a cell or being endogenously expressed, but because these genes are enriched in other cell populations within the dataset, DecontX probabilistically assigns them as cross-cluster contamination.

### Analysis of RBC and PLT transcripts in relation to MT-reads content

**Fig. 3:**
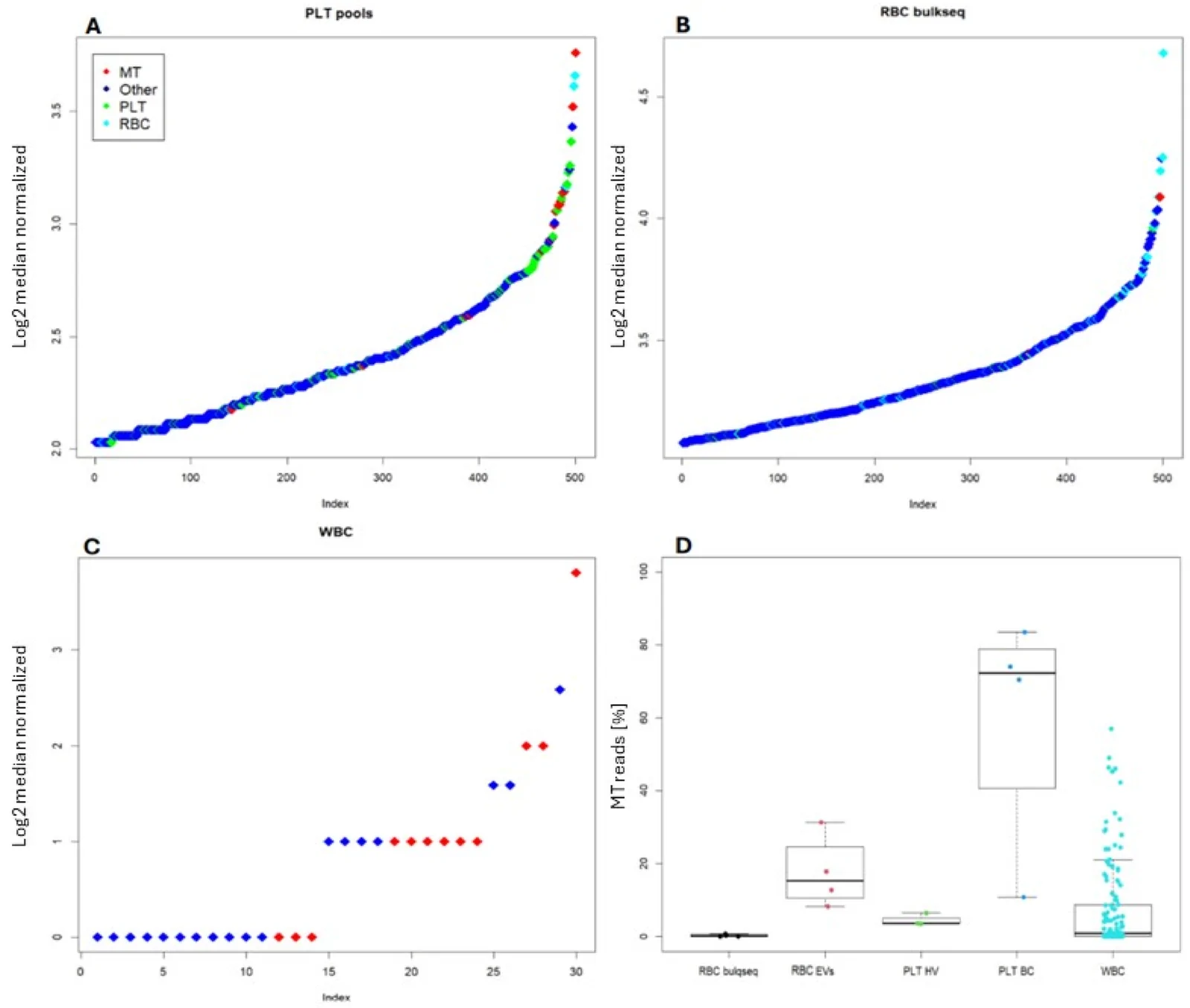
Expression of PLT, RBC markers and MT-reads in PLT, RBC, and WBCs reference samples. Median (log_2_) expression level of the top 500 genes expressed in **A** PLT pools, **B** RBC bulk sequencing, **C** in single WBCs with median gene expression > 0. **D** percentage of MT**-**reads in groups of samples (bulk sequenced RBCs, RBC-derived EVs, PLTs of health volunteers (HV), PLT from BC patients, as well as WBC)

**Fig. 4:**
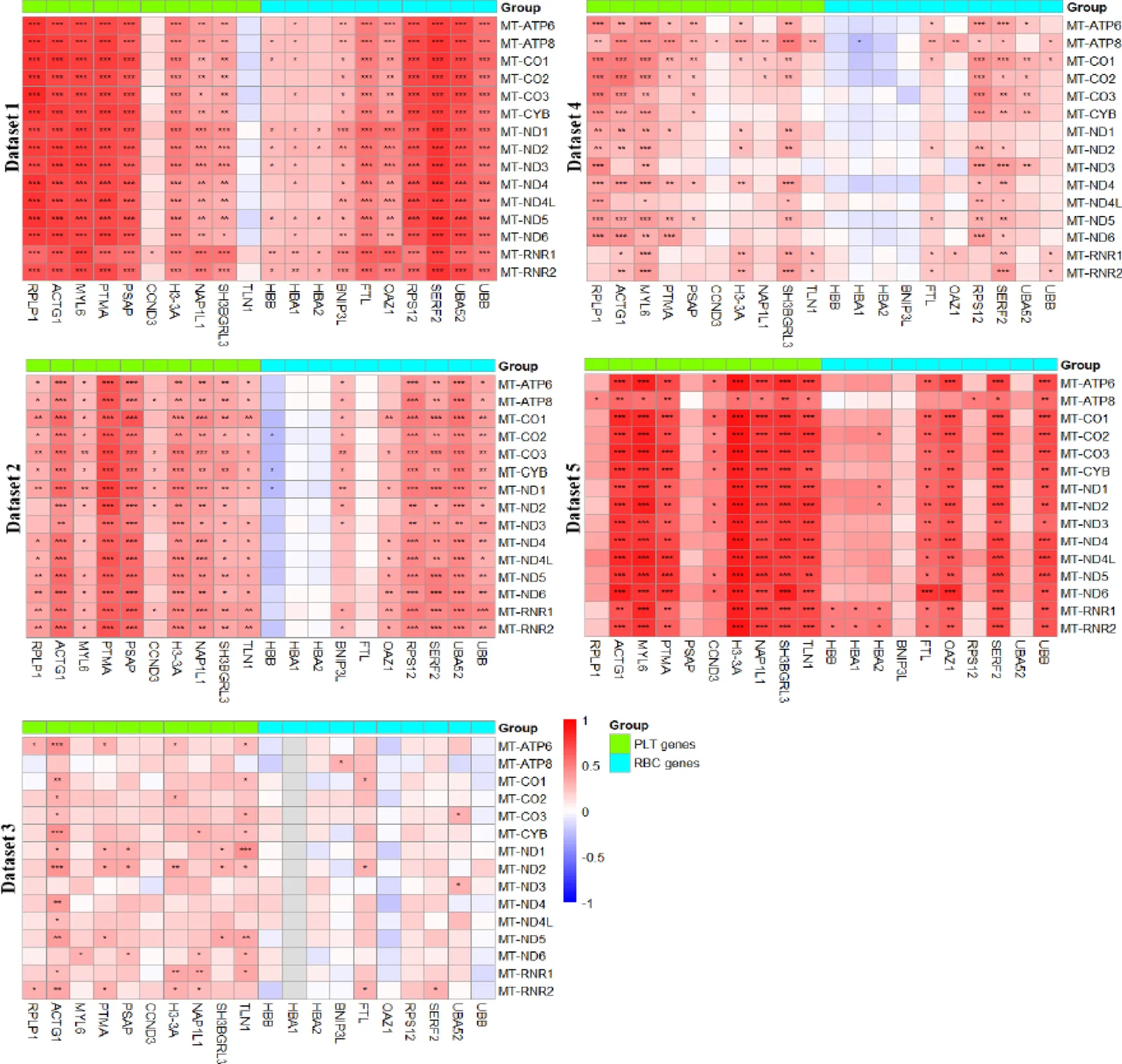
Correlations between PLT andRBC markers as well as MT-encoded genes expression in CTCs. Spearman rank correlation coefficients between expression level of selected marker genes of PLT (green), RBC (light blue) and MT-encoded genes in single CTCs from atasets 1–5. *p < 0.05. ** *p* < 0.01, *** *p* < 0.001

**Fig. 5:**
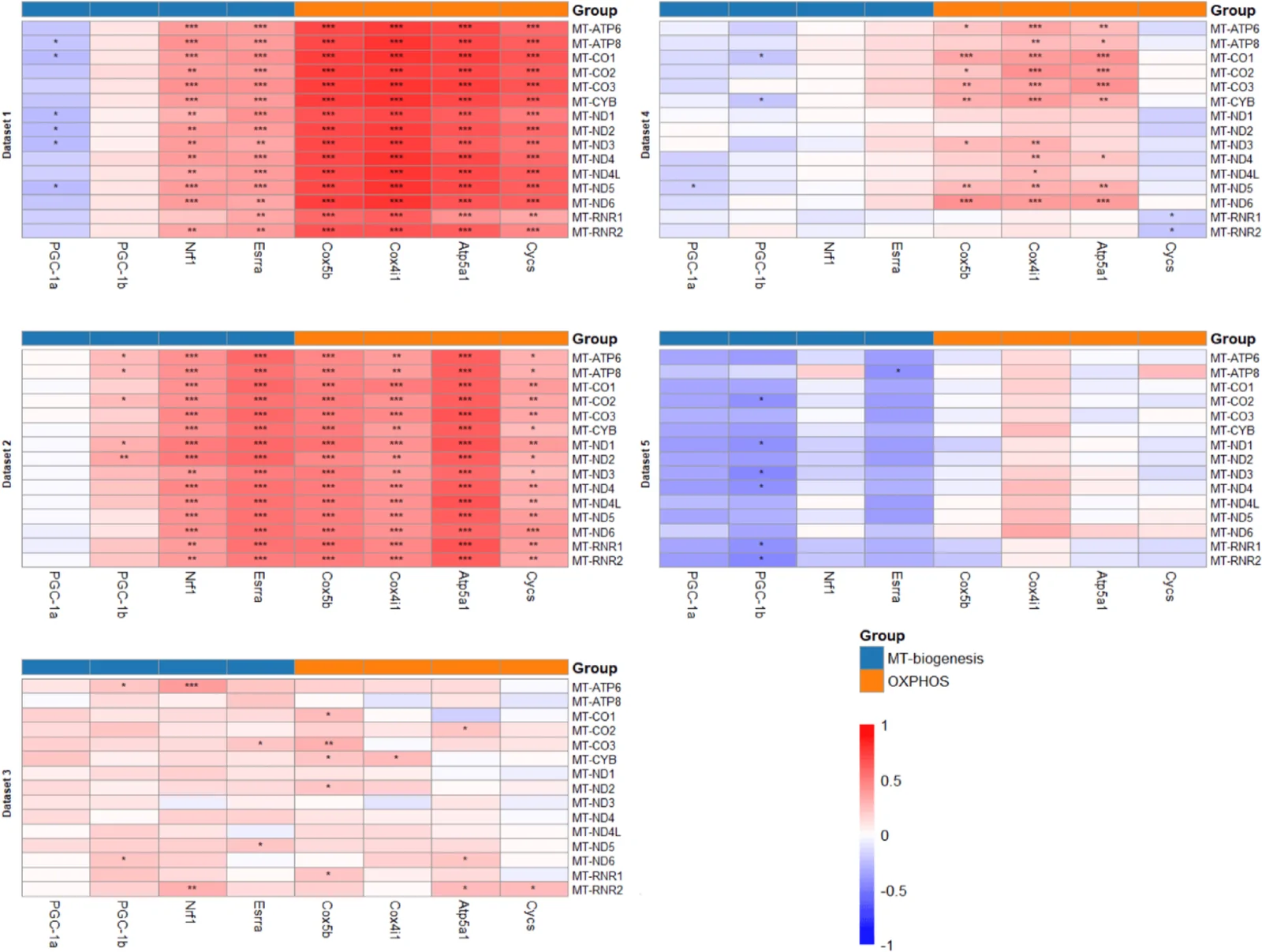
Correlations between MT-encoded mitochondrial biogenesis (MT-biogenesis) and oxidative phosphorylation (OXPHOS) genes in CTCs. Spearman rank correlation coefficients from datasets 1–5. *p < 0.05. ** *p* < 0.01, *** *p* < 0.001

As most CTCs show expression of PLT and RBC markers as well as MT-reads, we analysed the transcriptomes of PLT pools, RBCs, and WBCs to check the abundance of MT-reads and cell-type specific marker genes. In PLT samples (from 4 cancer patients and 3 healthy volunteers, each containing around 100 PLTs), the most highly expressed genes were MT genes (*MT-RNR2* coding for 16S rRNA and *MT-RNR1* coding for 12S rRNA) and RBCs genes (*HBB* coding for β-globin and *HBA2* coding for α-globin subunit 2, Fig. 3A, Supplementary Table 4). This indicates the presence of erythroid-associated transcripts within platelet pools, consistent with intercellular RNA exchange or transcript carryover [25, 72]. Interestingly, the percentage of MT-reads was considerably higher in PLTs from BC patients (mean 59.71%, SD 33.02%) than in PLTs from healthy volunteers (mean 4.57%, SD 1.72%), through difference did not reach conventional statistical significance (Wilcoxon rank-sum test, *p* = 0.052) and should therefore be interpreted as an exploratory observation given the limited sample size. As expected, *HBB* and *HBA2*, genes characteristic of RBCs, were most highly expressed in RBC bulk samples (*n* = 4), though mitochondrially encoded 16S rRNA (*MT-RNR2*) was the 5^th^ top expressed gene (Fig. 3B, Supplementary Table 4). Also, in RBC-derived EVs (*n* = 4), MT-encoded genes were highly represented with a median of 15% of all reads (Fig. 3D). MT-encoded genes were also highly expressed in WBCs, and at the same time PLT and RBC markers were not detected in the top 30 expressed genes of WBC (Fig. 3C). Correlating the expression level of selected highly expressed RBC and PLT genes (from Supplementary Table 4) with MT-encoded genes (protein-coding and rRNA) in single CTCs from each dataset, we observed mostly positive associations, especially for the PLT-genes (Fig. 4). These findings are consistent with the possibility that PLT-derived transcripts contribute to the MT-reads abundance observed in single CTCs. RBC-genes showed more heterogenous and dataset-specific correlations. In two datasets, (1 and 5) main RBC-markers - globin genes *HBB*,* HBA1*,* HBA2* were positively correlated with selected MT-reads. In ataset 2 and 4 other RBC-expressed genes (e.g., *UBB*,* UBA52*,* SERF2*) were positively correlated with the level of MT-reads. Similar associations were observed for single WBC (Supplementary Fig. 4). In that analysis we can not differentiate whether RBC and PLT transcripts could originate from RBC or PLT themselves or their EVs; RBC-derived EVs have higher level of MT-reads (as shown in Fig. 3D), thus their contribution to MT-reads level in CTCs could be stronger, though experimental validation would be required. Alternatively, elevated MT-reads in CTCs could result from increased oxidative phosphorylation (OXPHOS) and mitochondrial biogenesis [73]. In the datasets 1, 2, and 4 MT-reads correlated with increased OXPHOS genes encoded in the nuclear genome (*COX5B*,* COX4I1*,* ATP5A1*,* CYCS)*, suggesting higher cellular energy demand (Fig. 5). Genes connected to mitochondrial biogenesis (*PGC-1 A*,* PGC-1B*,* NRF1*, *ESRRA)* correlated with MT-reads in atasets 1, 2, and to some extent 4, but not in dataset 3 and 5.

### UMAP clustering of CTCs and WBCs

**Fig. 6:**
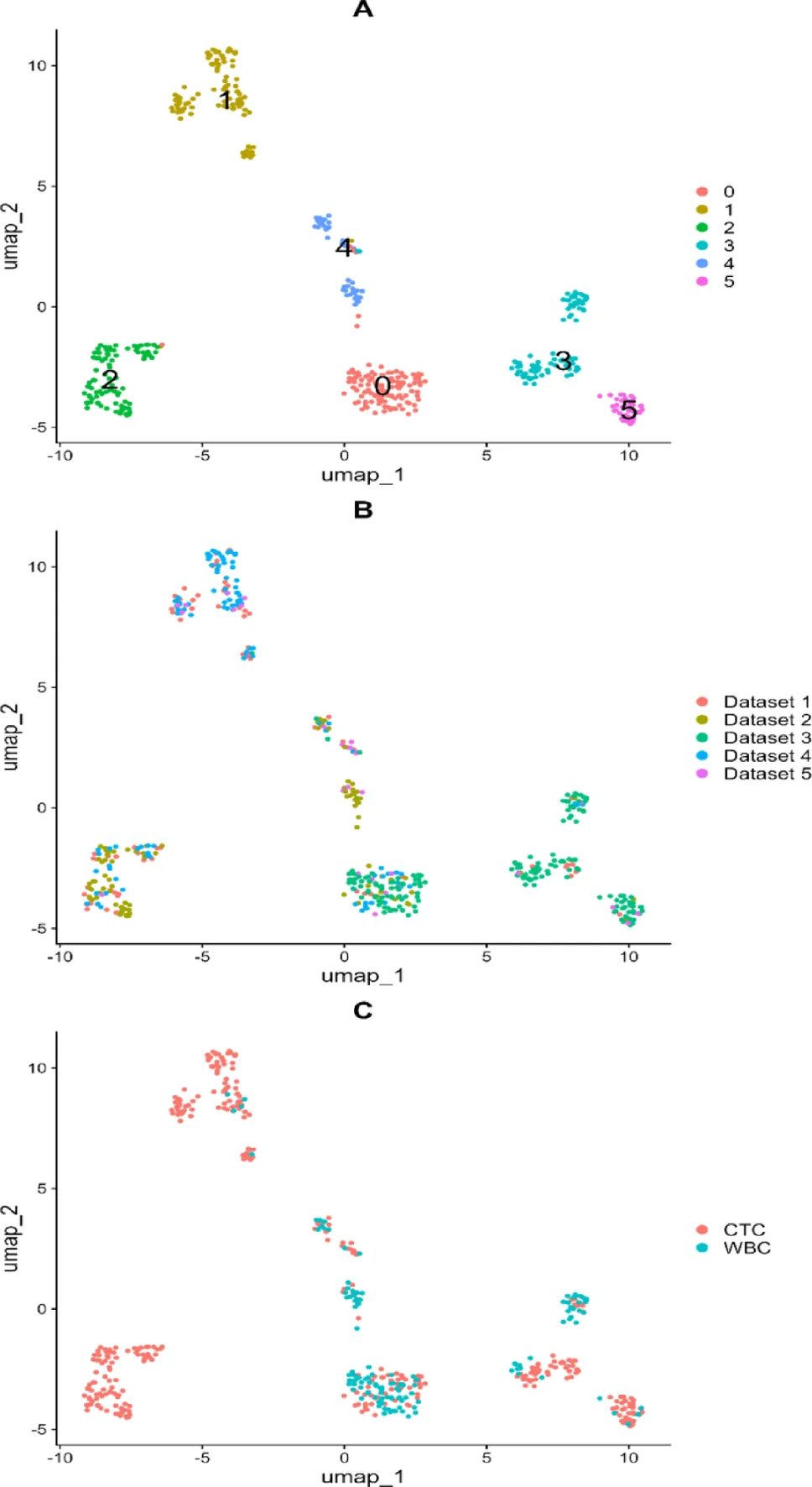
Integrated UMAP-based analysis of all processed CTCs and WBCs across the five datasets. **A** Unsupervised clustering of all single-cell transcriptomes after Harmony-based batch correction revealed six major transcriptionally distinct clusters (0–5), **B** UMAP colored according to the dataset of origin, demonstrating that cells from independent studies intermix within individual clusters, indicating successful integration and the presence of conserved transcriptional programs across datasets despite differences in experimental protocols and CTC enrichment methods, **C** Clusters 1, 2, and 5 contained the highest proportion of cells isolated based on defined protein-level characteristics of CTCs (see Table 1 in the manuscript)

**Fig. 7:**
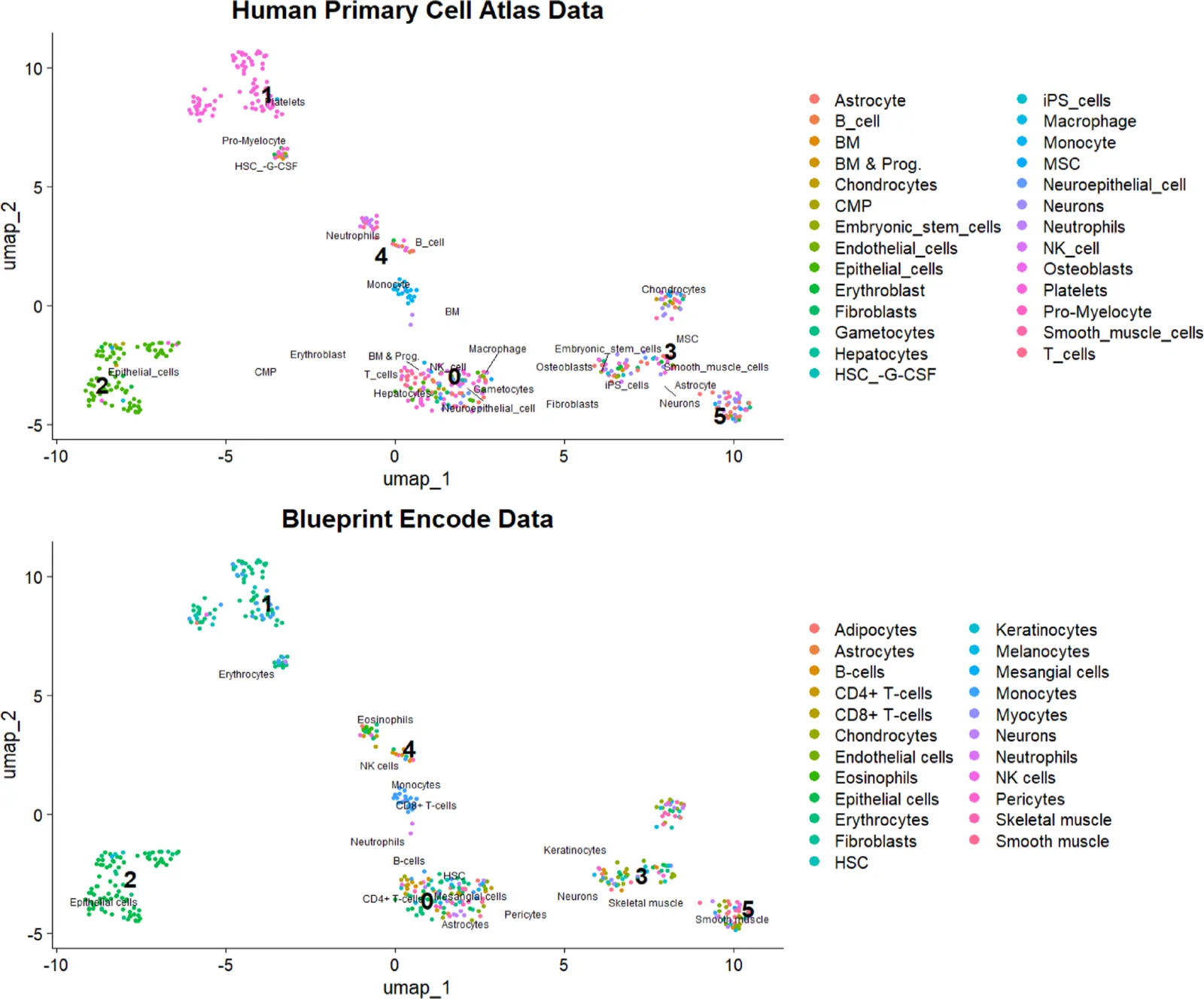
Reference-based annotation of integrated CTCs and WBCs transcriptomes using external human cell atlases. **A** Human Primary Cell Atlas (HPCA) and **B** Blueprint/ENCODE

**Fig. 8:**
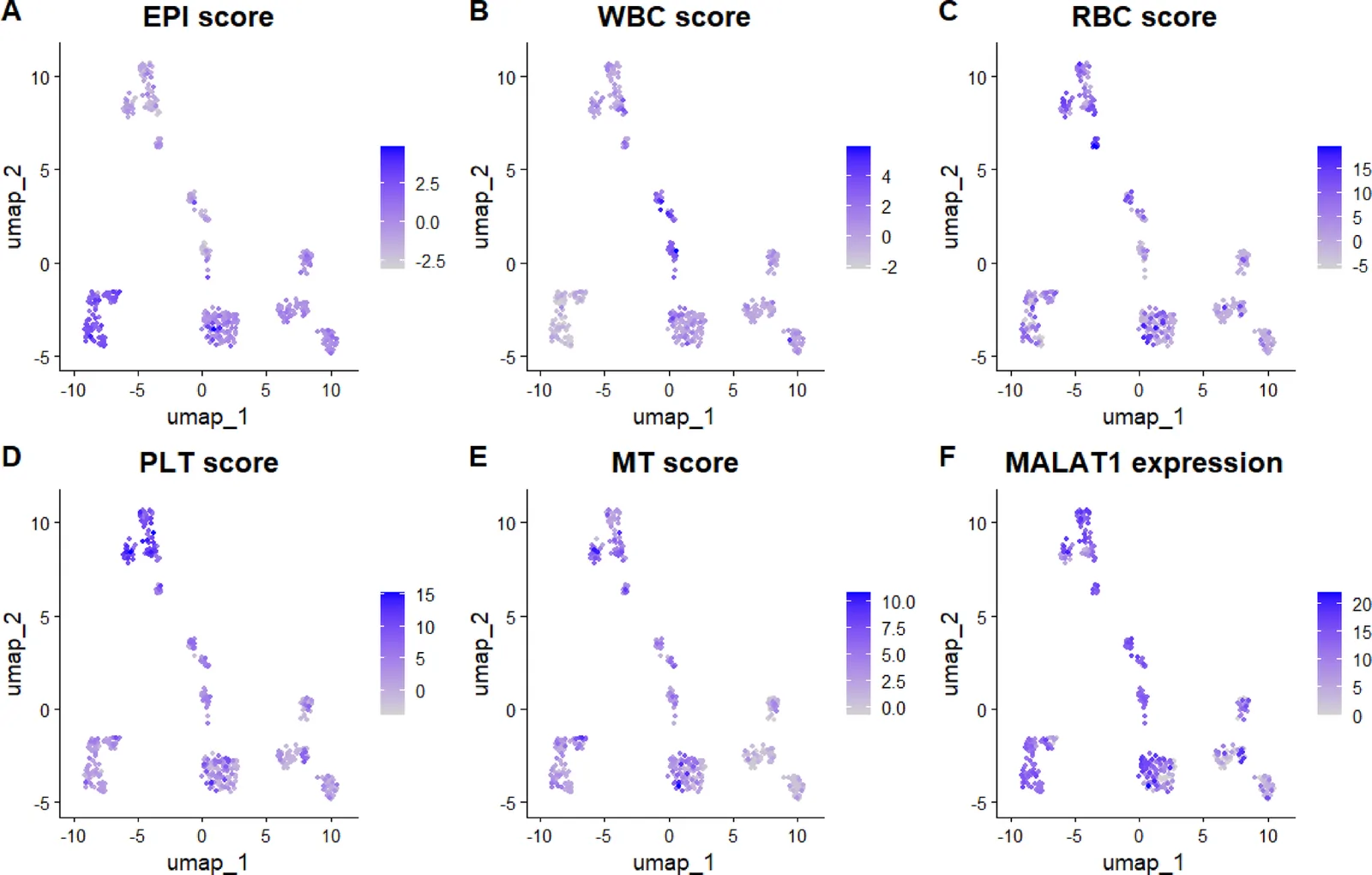
Cell types markers and mitochondrial-reads scores in CTCs and WBCs UMAP clusters. **A** Epithelial cell score (composed of *CDH1*,* EpCAM*,* KRT8*,* KRT18*,* KRT19*,* KRT5*,* KRT6A*,* KRT6B*,* KRT14*), **B** White blood cells score (composed of *PTPRC*,* CD3D*,* CD14*,* CD19*,* GZMA*), **C** red blood cells score (composed of *HBB*,* HBA1*,* HBA2*), **D** platelets score (composed of *NAP1L1*,* TLN1*), **E** MT-score (composed of 37 mitochondrially-encoded genes), **F** *MALAT1* expression. Expression of individual genes in the scores is presented in Supplementary Fig. 5

Because the five atasets were generated across independent cohorts and workflows, we performed joint integration and unsupervised UMAP clustering of all cells to minimize dataset-specific technical effects. This cross-dataset harmonization enabled identification of conserved transcriptional programs and detection of potential contaminating populations, providing additional validation of CTC identity beyond surface marker–based enrichment. UMAP clustering of all CTCs and WBCs from five atasets revealed six main clusters, with samples from different datasets mixing between the clusters, what underscores shared transcriptional programs despite technical and biological differences between datasets (Fig. 6A–C). Clusters 0, 3 and 4 had the highest fraction of WBCs (as annotated in the original datasets) (Fig. 6C) among which cells picked as CTCs (as annotated in the original datasets based on presence of surface protein markers) were also found. UMAP clustering projected onto Human Primary Cell Atlas labels and Blueprint/ENCODE (Fig. 7), revealed one clear epithelial cell cluster (cluster 2 of the original UMAP). Interestingly, cluster 1 was identified as platelets (Fig. 7—Human Primary Cell Atlas) or erythrocytes (Fig. 7—Blueprint/ENCODE) confirming significant contribution of PLTs and RBCs transcripts in the isolated single cells. We acknowledge the inherent limitations of reference atlas–based cell type annotation approaches, particularly when applied to single-cell RNA sequencing data, and therefore these results should be interpreted with caution. Nevertheless, consistent with Human Primary Cell Atlas and Blueprint/ENCODE cell labels were expression level-derived scores (Fig. 8)—EPI score (*CDH1*,* EpCAM*,* KRT8*,* KRT18*,* KRT19*,* KRT5*,* KRT6A*,* KRT6B*,* KRT14*), WBC score (*PTPRC*,* CD3D*,* CD14*,* CD19*,* GZMA)*, PLT score (*NAP1L1*,* TLN1*), RBC score (*HBB*,* HBA1*,* HBA2*), and MT score (all 37 mitochondrially-encoded genes). In cluster 2 we observed cells with high EPI score and low WBC score (Fig. 8A B); PLT and RBC scores were enriched in cluster 1 (Fig. 8C, D). MT-reads showed certain enrichment in cluster 1 together with PLT and RBCs scores (Fig. 8C D E). *MALAT1* expression, which is described as quality metrics in scRNA-seq informing about retention of nuclear transcripts [68] showed high expression in many UMAP clusters, including those with high PLT, RBC, and MT scores (Fig. 8F). WBC score was most prominent in cluster 4 and 0, that is where different immune cells labels by the Human Primary Cell Atlas and Blueprint/ENCODE (Fig. 7) were assigned and where WBC (as defined during micromanipulatory cell picking) were mostly found (Fig. 6C). Expression of individual genes from the EPI, WBC, RBC, PLT and MT scores is presented in Supplementary Fig. 5, with accompanying statistics in Supplementary Table 5.

**Fig. 9:**
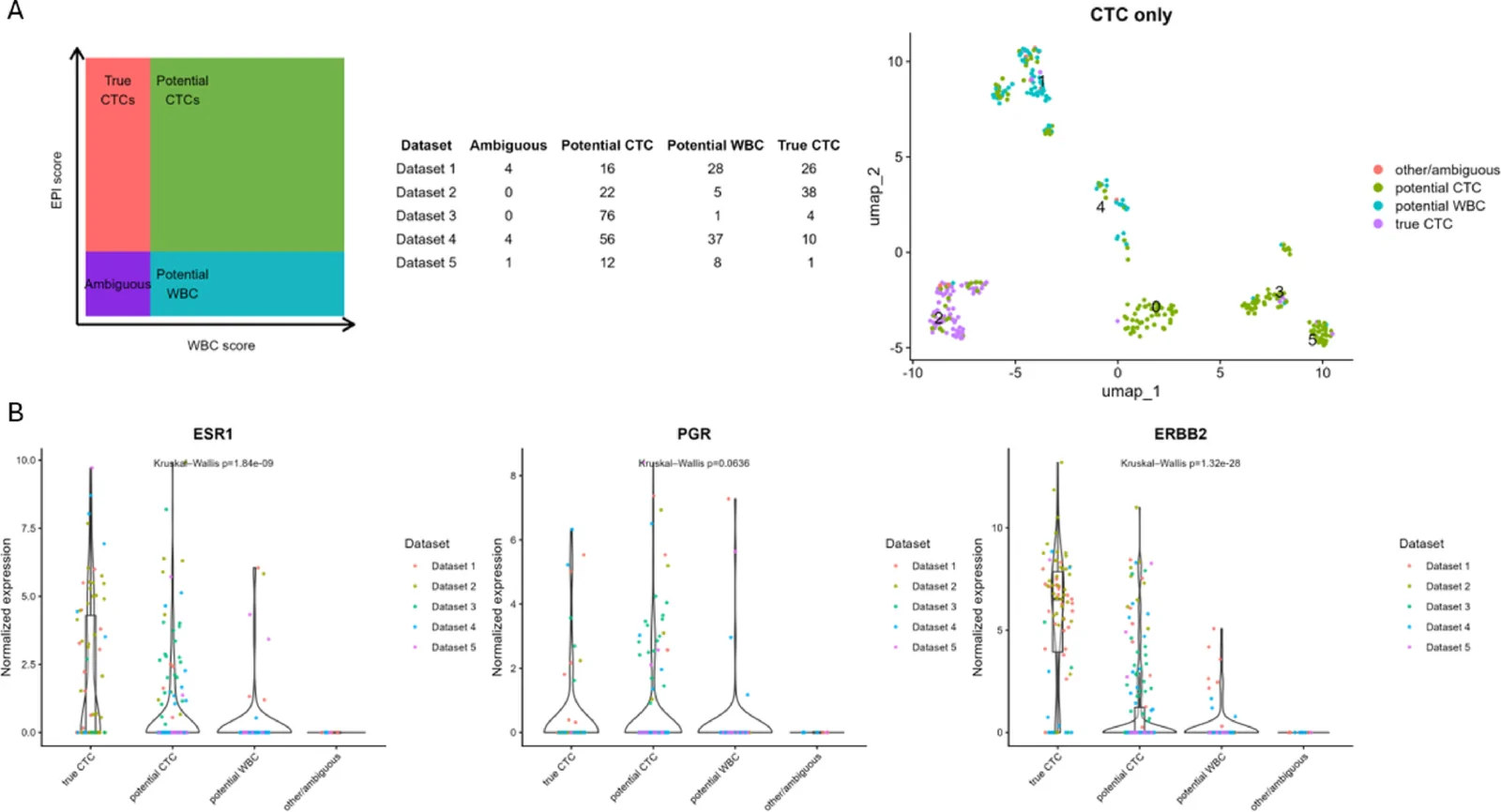
Classification of CTCs based on epithelial and white blood cells markers. **A** in order to define identity of the CTCs isolated in all studies, we divided CTCs based on epithelial markers expression (EPI score) and WBC markers expression (WBC score) to four groups based on given quartiles. Overlaid on the original UMAP clustering, ‘true CTCs’ class was enriched in cluster 2. Cluster 1 contained the most of ‘potential WBC’ and clusters 0, 3, and 5 contained mostly ‘potential CTCs’, whereas cluster 4 had a mixture of all classes, **B** breast cancer markers (which were not part of the EPI score)—*ESR1* and *ERBB2* (HER2), but not *PGR* showed increased expression especially in the ‘true CTCs’ group. The y-axis shows the log-normalized expression

**Fig. 10:**
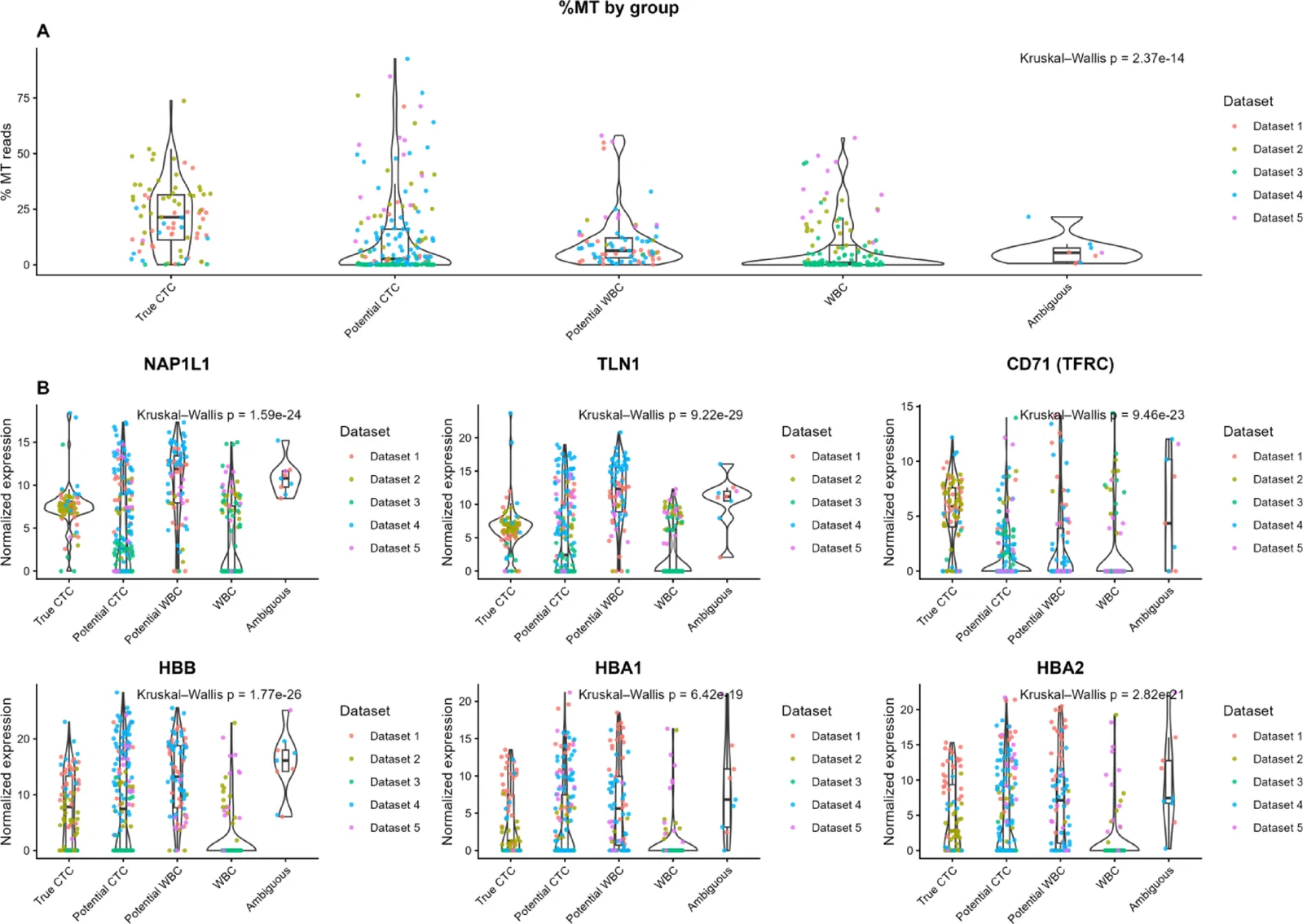
Level of MT-reads, PLT and RBC markers in CTCs and WBCs. Cells picked as CTCs were divided to four group (‘true CTCs’/’potential CTCs’/’potential WBCs’/’ambiguous’) according to EPI and WBC markers on Fig. 9A, **A** percentage of MT-reads, **B** expression of PLT markers (*NAP1L1*,* TLN1*), RBC (*HBB*,* HBA1*,* HBA2*) and reticulocytes marker (*CD71*). The y-axis shows the log-normalized expression

To delve deeper into biology and identity of cells isolated and annotated as CTCs according to the criteria defined in the individual datasets, we divided these CTCs based on epithelial markers expression (EPI score) and WBC markers expression (WBC score) to four groups (‘true CTCs’/’potential CTCs’/‘potential WBCs’/’ambiguous’) based on quartiles as shown in Fig. 9A. Cells from the ‘true CTC’ class were found across all datasets, and were especially enriched in the UMAP cluster 2 (Fig. 9A). ‘True CTCs’ class also showed the highest expression of different BC markers of luminal and HER2-positive subtype—ER (*ESR1* gene) and HER2 (*ERBB2* gene; Fig. 9B), Interestingly, ‘true CTCs’ class had the highest percentage of MT-reads (cells from dataset 2 showing highest expression of all; Fig. 10A), with low-intermediate levels of PLT markers (*TLN1*,* NAP1L1*), RBC markers (*HBB*,* HBA1*,* HBA2*), but higher expression of CD71 (Fig. 10B). CD71 is a transferrin receptor, expressed by erythroid progenitor cells [74, 75]. However, CD71 can also be expressed by BC cells, especially those with high energy demands [76, 77]. Higher expression of the CD71 in the ‘true CTC’ group (which also shows high levels of epithelial and BC markers gene expression) is consistent with elevated expression of mitochondrial biogenesis and OXPHOS genes (Fig. 5), as increased iron demand would be needed for assembly of iron–sulphur clusters and heme-containing electron transport chain complexes, thereby supporting elevated mitochondrial oxidative phosphorylation [78]. These observations are based on transcriptomic associations and will require experimental validation, ideally using CTC-derived models, to confirm the proposed biological mechanisms.

### Identifying genomic aberrations from transcriptomic profiles of CTCs

**Fig. 11:**
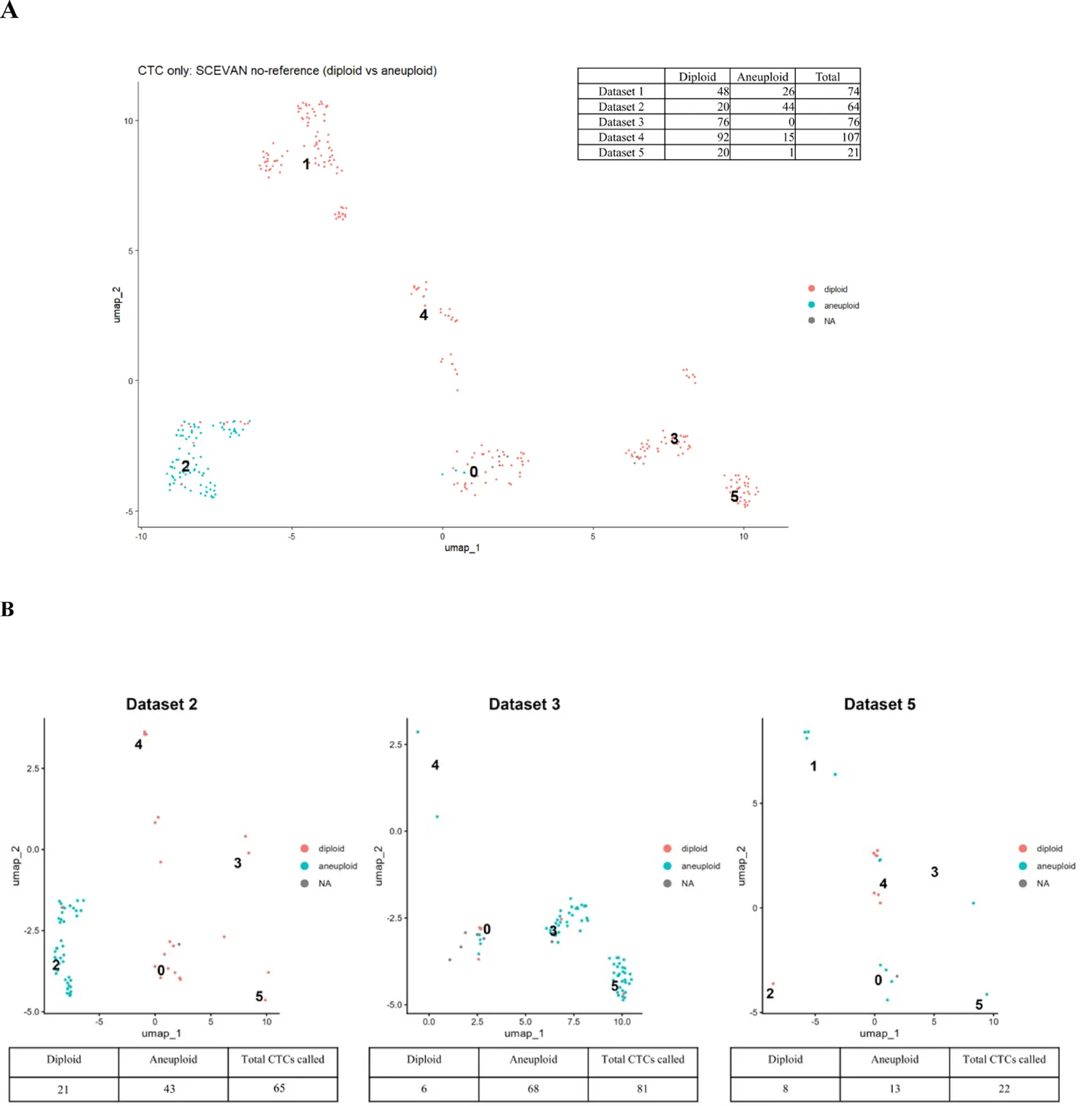
Inference of large-scale copy number alterations in CTCs. Cells picked as CTCs in individual datasets were classified as diploid or aneuploid using SCEVAN (**A**) or CopyKAT (**B**). For selected cells algorithms could not assign a class (NA). CopyKAT is shown for three datasets only as it uses WBC from the same dataset as an internal reference

To orthogonally check malignant identity of cells isolated and annotated as CTCs according to the criteria defined in the original studies, we looked at large-scale copy number alterations (which are associated with malignant cell origin) using single-cell transcriptomic CNV inference tools—SCEVAN and CopyKAT (Fig. 11). Based on SCEVAN aneuploidy criterion, 25% (86/342) of the analysed CTCs were classified as aneuploid and thus represent a conservative estimate of bona fide tumour cells (Fig. 11A). Consistently with high EPI score in UMAP, cells from cluster 2 exhibited the highest degree of inferred aneuploidy. It should be noted, however, that SCEVAN classification relies on transcriptional profiles and may assign cells with expression patterns resembling blood-derived populations to the diploid category. Moreover, mixed or contaminated expression signatures may reduce the sensitivity for detecting tumour-associated aneuploidy. Using CopyKAT, we assessed the ploidy status of CTCs in three datasets, for which WBC population as a diploid reference was available (Fig. 11B). CopyKAT identified additional aneuploid cells in atasets 3 and 5, resulting in a total of 73.8% (124/168) of cells being classified as aneuploid. Importantly, the absolute proportions of cells classified as aneuploid should not be directly compared across datasets, as differences in CTC pre-selection criteria, patient disease stage may influence genomic aberration burden of the analysed cell populations.

### Differentially expressed genes between CTCs and WBCs

To better understand the biology of cells isolated and annotated as CTCs according to the criteria defined in the original studies , we compared their gene expression profiles with WBCs in order to identify the most differentially expressed genes (DEGs) between these two groups using two approaches; in the first approach cells annotated in the original atasets (2, 3, and 5) as CTCs were compared with cells annotated as WBCs in the same datasets (Supplementary Table 6.1). Interestingly, 10 out of the 20 top DEGs were MT-encoded transcripts, 7 of which (*MT-RNR2*,* MT-ND4*,* MT-CO1*,* MT-CO2*,* MT-CYB*,* MT-ND5*, and *MT-ND6*) had an FDR < 0.05. This represents a significant enrichment of MT genes in CTCs, given that the MT genome encodes only 37 genes in total. Among the top DEGs, we also identified genes associated with cell adhesion, which could be potential markers for CTCs detection. For example, LIM domain-containing preferred translocation partner in lipoma (LPP) is involved both in focal and cell-cell adhesion [79], and Pals1-associated tight junction (PATJ) protein is associated with epithelial cell tight junctions [80]. In the second approach we included only cells from dataset 2 as in this dataset the highest fraction of cells showed features of malignant epithelial phenotypes (aneuploidy—68.7% according to SCEVAN, 66.2% according to copyKAT; ‘true CTC’ signature EPI-high and WBC-low score—58% of cells). ‘True CTCs’ from dataset 2 were compared with WBCs from the same dataset. Similarly, to the whole CTCs versus WBCs comparison (Supplementary Table 6.1), the top DEGs were MT-encoded transcripts (Supplementary Table 6.2), indicating that MT genes enrichment is a shared and robust characteristic of CTCs transcriptomes identified in both analyses.

## Discussion

Single-cell transcriptomics is a novel and powerful approach with the potential to revolutionize cancer research [81], enabling the detailed exploration of cellular heterogeneity within tumours and their environments [82]. A unique branch of scRNA-seq studies is the analysis of extremely rare cells, such as CTCs. In this study we analysed transcriptomic data of CTCs isolated in different laboratories using four different methods (Table 1, Supplementary document1). In two studies (atasets 1 and 4), the enrichment step was performed using the microfluidic CTC-iChip, enriching CTCs based on size, depletion of immune cells based on surface markers and further staining of CTCs’ protein markers (EpCAM, HER2, and additionally Cadherin 11 in dataset 4). Dataset 2 included CTCs isolated with Parsortix^TM^ microfluidic device, which takes advantage of CTCs’ larger size and lower deformability than WBCs. In dataset 3 RBC lysis was followed by enrichment with ClearCell^®^ FX, which is a spiral microfluidic system for label-free separation of CTCs from blood cells. In our dataset 5, CTCs were enriched by density gradient centrifugation, followed by immunofluorescent staining (EpCAM, E-cadherin, csVIM, MCAM, CD45, CD31) and depletion of CD45-positive cells with magnetic Dynabeads. In all studies mentioned above, except ataset 3, individual cells (CTCs and WBCs) were picked with a micromanipulator; in dataset 3 cells were collected using the ClearCell^®^ FX/Polaris^TM^ system based on their viability and lack of CD45 and CD31 expression. In scRNA-seq analyses, MT-reads content is commonly used as a quality indicator. However, the exact threshold for distinguishing low-quality cells is not well established, as it can vary significantly between organisms and tissues [11, 12]. Using a 10% MT-reads cut-off, a large proportion of CTCs scRNA-seq data would be considered low-quality and potentially rejected [12]. Our main question was whether MT-reads content accurately reflects quality of CTCs. We observed that MT-reads level in CTCs frequently correlates with expression of RBCs and PLTs markers, and at the same time both PLTs and RBCs transcriptome showed high level of MT-reads. Moreover, transcriptome complexity and MT-reads fraction was positively correlated with *MALAT1* expression, suggesting that MT-reads are not a standalone measure of poor transcriptome quality. Based on the experimental data from other studies we can only speculate on the potential mechanisms; we hypothesized that, at least to some degree, MT-reads detected in CTCs might be explained by contribution of transcripts from PLTs, RBCs, or PLT/RBC-derived EVs. This possibility seems particularly relevant for PLTs, which contain approximately 4–10 mitochondria per single PLT [31, 83] and PLTs themselves were shown to cooperate with cancer cells [29], creating a physical shield around CTCs [18]. Also, extracellular fragments, such as EVs, might mediate mitochondria transfer. PLT-derived EVs are the most abundant EVs in blood [84], and they were shown to increase in number during cancer development [85]. Interestingly, according to Zhang et al., PLTs are also capable of releasing whole mitochondria, which are captured by cancer cells and increasing their metastatic potential [33]. This interaction between cancer cells and PLTs or their mitochondria, might explain why MT-encoded genes were among the most differentially expressed genes between CTCs and WBCs. Furthermore, it is well known that many aspects of PLT biology are modified in cancer patients, including the clotting process [86], in which mitochondria are involved [27]. Regarding RBC, it is known that mature RBC do not contain mitochondria [87], though they might still retain some mitochondrial transcripts [88] or they can act as scavengers of mtDNA [89] released from dying cells. On the other hand, higher MT-reads or erythroid signature in CTCs may be biologically acquired. Zheng et al. [71] demonstrated endogenous β-globin expression in CTCs as a consequence of elevated reactive oxygen species levels, whereas LeBleu et al. [73] detected enhanced oxidative phosphorylation and mitochondria biogenesis in CTCs. Moreover, as shown by Sansone et al. EVs-mediated mitochondrial genome uptake by cancer cells increased their level of oxidative phosphorylation [69]. Consistent with these results, was our observation that cells characterized by high EPI-score and low WBC-score (‘true CTCs’) had a higher level of MT-reads and displayed elevated expression of genes associated with oxidative phosphorylation and mitochondrial biogenesis. We observed differences in MT, PLT, and RBC reads levels in different datasets. Interestingly, CTCs from two different datasets, but enriched with the same method, i.e., CTC-iChip (atasets 1 and 4), exhibited a similar level of marker genes contribution (RBC and MT-reads), possibly indicating a method-specific pattern. Moreover, it is conceivable that some methods/protocols of CTCs enrichment are more likely to cause cell damage or bear a higher risk of collecting ambient transcripts, resulting in high levels of MT or other cell type-lineage markers. CTCs isolated with Parsortix (dataset 2) did not exhibit a high proportion of RBCs or PLTs transcripts but did contain the highest percentage of MT-reads, which distinguishes them from the other datasets. GSEA have not identified enrichment in apoptotic signature in CTCs from dataset 2 (though WBCs from the same dataset did), which might indicate other mechanisms of transcripts acquisition (endogenous or exogenous). The origin of the transcripts characteristic for blood cell types (RBC and PLTs) detected in CTCs should be further investigated to establish to what extent technical artifacts are playing a role and when actual interactions or biological CTCs-endogenous processes take place. CTCs circulate in close contact with highly abundant blood components, particularly PLTs and RBCs, and may acquire RNA through vesicle-dependent or vesicle-independent transfer mechanisms. Extracellular RNA can be transported in ribonucleoprotein complexes [90–92] or encapsulated in extracellular vesicles [93, 94]. Also, collection of a single CTC together with PLTs might occur during CTC picking, especially when a CTC is covered with PLTs. In addition, RNA released from stressed, damaged, or dying blood cells during sample collection and processing may contribute to transcript admixture [57, 95]. PLTs may also be activated and release into the sample processing buffer the content of their granules, which might contain RNAs [96, 97]. As a result, both the transcriptome of the CTCs and ambient RNA present in the solution might be profiled during RNA-Seq. We can not distinguish between these two non-mutually exclusive sources of PLT and RBC markers in CTCs - exogenous (technical artefacts linked to presence of ambient RNA) and endogenous sources (biologically meaningful processes like true gene expression in CTCs or active uptake of RNAs). Experimental studies aiming at quantification of both technical and biological processes during CTCs analysis using different platforms would be required for accurate assessment of mechanisms contributing to MT-reads, PLT, RBC markers in CTCs.

Cells isolated and annotated as CTCs may display characteristics typically associated with cancer cells, such as epithelial marker expression, absence of hematopoietic markers, or increased cell size, yet these features alone do not unequivocally confirm their malignant origin. Conversely, bona fide CTCs could exhibit expression of lineage-associated transcripts originating from cellular plasticity or interactions with surrounding blood cells, complicating their identification. Erythroid progenitors present in the bone marrow, as well as CD34^+^ cells detectable in peripheral blood, may express epithelial markers such as EpCAM [36, 98, 99]. Ectopic expression of epithelial markers by non-epithelial cells (and their false-positive classification as CTCs) does not exclude the scenarios that would lead to false-negative classification of CTCs due to (1) CTCs inherently expressing markers of other cell lineages or (2) carryover of different blood cell transcripts to CTCs. Gužvic et al. [37], showed the presence of genomic aberrations in DTCs with hematopoietic and erythroid-cell markers, proving that lineage marker expression in DTCs does not necessarily exclude malignant origin. Overall, these findings underscore the inherent limitations of reliably determining the cancerous origin of rare cells using restricted marker panels. To increase confidence in cells’ classification, we combined multiple complementary transcriptomic approaches, including extended panels of epithelial and leukocyte markers, reference atlas projection, and inference of large-scale copy number alterations using SCEVAN and CopyKAT. A subset of cells (Cluster 2 on the UMAP) consistently exhibited multiple independent features supporting malignant epithelial identity, including high epithelial marker expression, low leukocyte marker expression, annotation as epithelial cells by reference atlas projection, and inferred aneuploidy. However, the substantial differences between SCEVAN and CopyKAT, with considerably more cells classified as aneuploid by CopyKAT, underscore the limitations of CNV inference from sparse single-cell transcriptomes and demonstrate that no single computational approach can unequivocally establish malignant identity. Moreover, our results should not be used for direct comparison of absolute numbers of CTCs defined as aneuploid as some datasets used pre-selection criteria, removing cells expressing immune cells or stromal cells markers, which could affect the proportion of cells classified as aberrant. Also, stage of the disease could affect the number of aberrations detected—studies on disseminated tumour cells (DTC) have demonstrated that DTC from non-metastatic patients exhibit fewer genomic aberrations than those from metastatic patients [100, 101]. Consequently, the sensitivity of transcriptome-based CNV inference could vary across the analysed datasets as patients from whom samples were collected ranged from non-metastatic to metastatic.

Due to limited available information regarding sample collection and preanalytical steps, we can not assess to what extent the differences between the datasets were related to the method of CTCs enrichment/collection or other biological or pre-analytical factors, such as the time from sample collection to processing, blood sample storage temperature, tumour molecular subtype, or stage of the disease. In individual datasets, CTCs were obtained from patients with different disease stage (non-metastatic vs. metastatic) and treatment status (treated vs. non-treated), what represents an inherent limitation of the integrated analysis and may contribute to the biological variability among CTC transcriptomes and should be considered in cross-datasets comparisons. Therefore, experimental validation with direct comparison of the same samples processed with the original protocols described in the respective studies would be required to establish causality and to disentangle technical artifacts from biological signal.

To summarize, we showed that transcripts characteristic of PLT and RBC can significantly contribute to the transcriptomic profile of single CTCs. The median percentage of PLT and RBC marker transcripts in CTCs was 2% (range: 0–38%) and 0.8% (range: 0–82%), respectively, in all datasets. To our knowledge, this is the first study to demonstrate that the analysis of CTCs might require different standards in terms of evaluating the transcriptome quality by MT-reads. We observed associations between MT-reads and PLT, RBC marker genes as well as encoded by the nuclear genome transcripts indicative of mitochondrial biogenesis and oxidative phosphorylation. Our presumption (which requires experimental validation) is that MT-reads might arise from different sources—inherent changes occurring in cancer cells during metastasis (e.g., increased mitochondrial activity in CTCs), capturing transcripts from other cell types (such as PLTs and RBCs), or the level of ambient RNA. Therefore, MT-reads abundance might not be a reliable standalone quality metric for single CTC transcriptomes and should be interpreted together with additional quality and biological parameters of the single cells. These findings have important implications for methodological and translational aspects of CTCs-derived data interpretation. Future CTC RNA-seq analyses should systematically evaluate MT-reads and blood-derived transcript signatures (e.g., PLT/RBC markers) to improve interpretation of CTCs’ biology. Our results also demonstrate that transcriptomic profiles of single cells can potentially facilitate the evaluation of the performance of CTCs isolation methods and protocols, which might be helpful to further develop tools for the isolation of pure, good-quality CTCs. We hope that our results will be the starting point for a discussion on the formulation and implementation of guidelines that would allow to correct observed limitations in the field of CTC analysis.

## Supplementary Information

Below is the link to the electronic supplementary material.

Supplementary Material 1

Supplementary Material 2

Supplementary Material 3

## Data Availability

Single-cell RNA-seq data used in this study (Dataset 5) have been deposited in the Gene Expression Omnibus (GEO) repository under accession number GSE268201.The following publicly available datasets were also used: CTCs/WBCs – GSE75367, GSE109761, GSE210651, GSE186288, GSE143626, GSE268201; RBCs and RBC-EVs – GSE108378; PLTs – GSE109761.

## Abbreviations

BC: Breast cancer
CTCs: Circulating tumour cells
csVIM: Cell surface vimentin
EMT: Epithelial–to–mesenchymal transition
EGFR: Epidermal growth factor receptor
EpCAM: Epithelial cell adhesion molecules
ER: Estrogen receptor
EVs: Extracellular vesicles
HER2: Human epidermal growth factor receptor type 2
LPP: LIM domain–containing preferred translocation partner in lipoma
MALAT1: Metastasis associated lung adenocarcinoma transcript 1
MCAM: Melanoma cell adhesion molecule
MT: mitochondrial
OXPHOS: Oxidative phosphorylation
PLT: Platelets
PR: Progesterone receptor
PTPRC: Protein tyrosine phosphatase receptor type C (CD45–encoding gene)
RBCs: Red blood cells
scRNA-Seq: single–cell RNA sequencing
SD: Standard deviation
SRR file: Sequence Read Run file
TNBC: Triple–negative breast cancer
WBCs: White blood cells
