# Supplementary material for: Mitochondrial transcript abundance in breast cancer circulating tumour cells may reflect both blood-derived transcripts and biological heterogeneity
Source: Clin Exp Metastasis. 2026 Oct 5;43(5):52. doi: 10.1007/s10585-026-10430-0 (PMC13638734; doi:10.1007/s10585-026-10430-0)
Supplement: Supplementary file 1 [file 10585_2026_10430_MOESM1_ESM.docx]

**Mitochondrial transcript abundance in breast cancer circulating tumor cells may reflect both blood-derived transcripts and biological heterogeneity**

Alicja Staśczak^1,2^, Agnieszka Stankiewicz^3^, Justyna Topa^3^, Zofia Piesik^4^, Anna Żaczek^3^, Sebastian Student^1,2^, Tomasz Stokowy^5,6*^, Aleksandra Markiewicz^3*^

^1^Department of Systems Biology and Engineering, Faculty of Automatic Control, Electronics and Computer Science, Silesian University of Technology, Poland

^2^ Biotechnology Centre, Silesian University of Technology, Poland

^3^ Laboratory of Translational Oncology, Intercollegiate Faculty of Biotechnology, University of Gdańsk and Medical University of Gdańsk, Poland

^4^ Institute of Informatics, Faculty of Mathematics, Physics and Informatics, University of Gdańsk, Poland

^5^ Scientific Computing Group, IT Division, University of Bergen, Norway

^6^ Interdisciplinary Pomeranian Centre for Digital Medicine, Medical University of Gdańsk, Poland

**Supplementary Document 1**

**CTCs enrichment methods in the Datasets**

**Dataset 1**

Whole blood was labelled with biotinylated antibodies against immune cells CD45, CD66b, CD16, followed by incubation with Streptavidin-magnetic beads. Samples were then processed via CTCs-iChip allowing for separation of RBC, platelets via hydrodynamic cell sorting and magnetically labelled immune cells were then magnetophoretically depleted from unlabelled cells. The unlabelled cells (containing putative CTCs) were then stained for EpCAM, HER2 and immune cell marker CD45 and screened under the microscope. Single cells fulfilling the predefined criteria of CTCs were picked by micromanipulator and transferred to lysis buffer.

Jordan NV, Bardia A, Wittner BS, et al. HER2 expression identifies dynamic functional states within circulating breast cancer cells. Nature. 2016;537(7618):102-106. doi:10.1038/nature19328

**Dataset 2**

Full blood samples were used for enrichment of CTCs in Parsortix device (6.5 um cassette) followed by staining of EpCAM, HER2, EGFR and CD45 in solution. Cell suspension was screened under the microscope. Single cells fulfilling the predefined criteria of CTCs were picked by micromanipulator and transferred to lysis buffer.

Szczerba BM, Castro-Giner F, Vetter M, et al. Neutrophils escort circulating tumour cells to enable cell cycle progression. Nature. 2019;566(7745):553-557. doi:10.1038/s41586-019-0915-y

**Dataset 3**

Full blood samples were subjected to RBC lysis, followed by size based selection on a spiral chip of the ClearCell FX system. Following the enrichment, cells were stained with cell tracker/viability markers - CellTracker Orange (CTO), Calcein AM, and exclusion markers - CD45, CD31 and loaded on the Polaris device, which allowed capturing of cells with a given phenotype – single cells which were CTO+ and Calcein AM+ and CD45- and CD31- were selected to capture sites and subjected to single cells cDNA generation on the Polaris device.

Poonia S, Goel A, Chawla S, et al. Marker-free characterization of full-length transcriptomes of single live circulating tumor cells. Genome Res. 2023;33(1):80-95. doi:10.1101/gr.276600.122

**Dataset 4**

Whole blood was labelled with antibodies against CD45 and CD66b followed by incubation with Dynabeads MyOne Streptavidin T1 (Invitrogen) to achieve magnetic labelling of the immune cells. Samples were then processed via CTCs-iChip allowing for separation of RBC, platelets via hydrodynamic cell sorting and magnetically labelled immune cells were then magnetophoretically depleted from unlabelled cells. The unlabelled cells (containing putative CTCs) were stained for EpCAM, Cadherin 11, HER2, as well as immune cell markers CD45, CD14, CD16, then screened under the microscope. Single cells fulfilling the predefined criteria of CTCs were picked by micromanipulator and transferred to lysis buffer.

Ebright RY, Lee S, Wittner BS, et al. Deregulation of ribosomal protein expression and translation promotes breast cancer metastasis. Science. 2020;367(6485):1468-1473. doi:10.1126/science.aay0939

**Dataset 5**

Whole blood was subjected to platelets removal and density gradient centrifugation, followed by immunofluorescent staining of the collected PBMC fraction with EpCAM, E-cadherin, cell-surface vimentin, MCAM and exclusion markers – CD45, CD31, DAPI. Next, samples were incubated Dynabeads™ CD45-magnetic beads to remove immune cells; following the depletion samples were screened under the microscope. Single cells fulfilling the predefined criteria of CTCs were picked by micromanipulator and transferred to lysis buffer.

Topa J, Richert J, Stokowy T, et al. Characterizing epithelial-mesenchymal transition-linked heterogeneity in breast cancer circulating tumor cells at a single-cell level. Mol Oncol. 2025;19(12):3685-3705. doi:10.1002/1878-0261.70132
