# Supplementary material for: Mitochondrial transcript abundance in breast cancer circulating tumour cells may reflect both blood-derived transcripts and biological heterogeneity
Source: Clin Exp Metastasis. 2026 Oct 5;43(5):52. doi: 10.1007/s10585-026-10430-0 (PMC13638734; doi:10.1007/s10585-026-10430-0)
Supplement: Supplementary file 2 [file 10585_2026_10430_MOESM3_ESM.docx]

**Submission to:** Clinical & Experimental Metastasis

**Mitochondrial transcript abundance in breast cancer circulating tumour cells may reflect both blood-derived transcripts and biological heterogeneity**

Alicja Staśczak^1,2^, Agnieszka Stankiewicz^3^, Justyna Topa^3^, Zofia Piesik^4^, Anna Żaczek^3^, Sebastian Student^1,2^, Tomasz Stokowy^5,6*^, Aleksandra Markiewicz^3*^

^1^Department of Systems Biology and Engineering, Faculty of Automatic Control, Electronics and Computer Science, Silesian University of Technology, Poland

^2^ Biotechnology Centre, Silesian University of Technology, Poland

^3^ Laboratory of Translational Oncology, Intercollegiate Faculty of Biotechnology, University of Gdańsk and Medical University of Gdańsk, Poland

^4^ Institute of Informatics, Faculty of Mathematics, Physics and Informatics, University of Gdańsk, Poland

^5^ Scientific Computing Group, IT Division, University of Bergen, Norway

^6^ Interdisciplinary Pomeranian Centre for Digital Medicine, Medical University of Gdańsk, Poland

**Corresponding author:** Aleksandra Markiewicz – aleksandra.markiewicz@gumed.edu.pl

**Supplementary Fig.** **1** Transcriptomic composition of white blood cells (WBCs) according to classes of transcripts. (A) Percentage of mapped reads in WBCs, in relation to all counts in a given cell, according to transcript class: breast cancer (BC) markers, WBC markers, red blood cells (RBCs) markers, platelets (PLTs) markers, mitochondrially-encoded (MT) genes, and other genes (Other); each bar corresponds to a single WBC. Numbers on the graph indicate the dataset number (2, 3, 5). (B) Single WBCs’ transcriptome contamination level estimated using the DecontX in individual datasets. Category I-IV show purity from highest to lowest. (C) Proportion of doublets in collected WBCs datasets. (D) Expression of canonical white blood cell markers across WBC datasets. Violin plots show the distribution of log-normalized expression levels of representative WBC marker genes (*PTPRC, CD3D, CD14, CD19*, and *GZMA*) in WBCs derived from Dataset 2, 3 and 5. Each panel corresponds to one marker gene, and expression levels are shown for individual cells within each dataset.

**A**

**B**

**C**

**D**

**Supplementary Fig. 2** Read counts and number of genes expressed in single cells. Number of read counts for each circulating tumour cell (A) and white blood cell (B). Number of genes expressed in each circulating tumour cell (C) and white blood cell (D). A single bar is one cell.

**Supplementary Fig. 3** Evaluation of the relationship between mitochondrial transcript abundance, apoptosis-related transcriptional signatures, and transcriptome quality in CTCs and WBCs. (A) Gene Set Enrichment Analysis (GSEA) of the apoptosis signature in cells with low (<10%) and high (≥10%) MT-reads fraction. Analyses were performed for CTCs from Datasets 1, 2, and 4 and WBCs from Dataset 2; (**B)** Apoptosis module scores in MT-low (<10%) and MT-high (≥10%) CTCs and WBCs from all Datasets. Differences were assessed using the Wilcoxon rank-sum test; (C) Correlation between the percentage of MT-reads and the total normalized expression of non-MT genes in WBCs and CTCs from all Datasets. Each point represents a single cell. The blue line indicates the fitted linear regression with the 95% confidence interval (grey shading), while the inset reports the Spearman correlation coefficient (ρ), associated *p*-value, and the number of analysed cells (*n*); (D) Spearman correlation analysis between normalized *MALAT1* expression and transcriptomic quality metrics excluding MT genes, including the total normalized expression of non-MT genes (‘Counts no MT’), the number of detected genes with normalized expression >0 (‘Genes >0 no MT’), and the number of detected genes with normalized expression >5 (‘Genes >5 no MT’), analysed separately for WBCs and CTCs. (E) Distribution of normalized *MALAT1* expression (log10-transformed) in cells with low (<10%) and high ( ≥10%) MT-reads fraction, shown separately for WBCs and CTCs. Differences between groups were evaluated using the Wilcoxon rank-sum test.

**Supplementary Fig. 4** Correlations between PLT, RBC and MT genes in WBCs. Spearman rank correlation coefficients between selected marker genes of PLT (green), RBC (light blue) and mitochondrially-encoded genes in single WBCs from Datasets 2, 3 and 5. *p<0.05. ** p<0.01, *** p < 0.001

**A**

**B**

**C**

**D**

**E**

**Supplementary Fig.** **5** Expression of marker genes in CTC and WBC UMAP clusters. Gene set activity was quantified as module scores calculated using the AddModuleScore function implemented in Seurat. Shown are module scores for (A) epithelial genes; (B) white blood cell genes; (C) red blood cell genes; (D) platelet genes; (E) all mitochondrially encoded genes.
